# A non-destructive methodological framework for modernizing legacy clinical reporting systems for AI-driven pharmacoinformatics: a SAS case study

**DOI:** 10.3389/fphar.2026.1876207

**Published:** 2026-09-28

**Authors:** Jaime Yan

**Affiliations:** Department of Data Science and Analytics, Harrisburg University of Science and Technology, Harrisburg, PA, United States

**Keywords:** artificial intelligence, CDISC, clinical reporting systems, intermediate representation, non-destructive modernization, pharmacoinformatics, pharmacovigilance, SAS

## Abstract

Legacy clinical reporting pipelines bottleneck drug development and pharmacovigilance: they encode regulatory-grade analytical logic but produce opaque output with no machine-readable intermediate layer, resisting integration with artificial intelligence (AI). Existing modernization approaches force a choice between full rewrites that abandon validated logic and incremental refactoring that preserves structural barriers to AI access. We present a non-destructive framework that achieves AI-driven pharmacoinformatics readiness without altering legacy source code. A metadata layer — a bridge map, a typed Intermediate Representation (IR), and an orchestrator — wraps existing reporting components, re-exposes their outputs as structured data consumable by large language models (LLMs), and enables optional incremental consolidation of selected components while the remainder operates unchanged. On a 558-component industrial SAS reporting library (373,000 lines of code), the framework demonstrated immediate AI readiness under coexistence mode with the legacy library unchanged. Where consolidation was elected, the modernized core achieved a 92% reduction in consolidated-core SAS lines of code (excluding the Python and YAML layers). Parity validation on 14 report types from an internal Phase III study cleared an 80% cell-level triage-admission gate on 11 of 14 reports (mean across all 14 report types 82.7%, best 99.2%); the three remaining reports differed in table-layout geometry rather than in computed values. A public benchmark on CDISC CDISCPilot01 data achieved 100% regression-consistency against frozen reference outputs across 5 reports (4,764 cells, 0 mismatches) — a reproducibility benchmark rather than independent validation. In a proof-of-concept study with a single large language model, the IR illustrated the feasibility of table summarization, adverse-event anomaly detection (a pharmacovigilance signal-detection example), and trial configuration generation — feasibility rather than validated performance. The framework offers a regulation-aware path to AI-integrated clinical trial reporting intended to reduce modernization overhead without interrupting ongoing regulatory submissions. Its regulatory-traceability features are design properties rather than validated compliance: no formal Installation/Operational/Performance Qualification (IQ/OQ/PQ) has been performed. All results derive from a single case study; the methodology is *designed to be* reusable across organizations and library scales, pending multi-site empirical validation.

## 1 Introduction

The pharmaceutical industry faces simultaneous pressure from three directions: AI tools now automate clinical data review and pharmacovigilance (Harrer et al., 2019), cloud-based platforms are replacing on-premise computing infrastructure (Griebel et al., 2015), and regulatory agencies increasingly require electronic submissions built on standardized data models (U.S. Food and Drug Administration, 2021). Together, these forces demand that clinical trial reporting infrastructure become machine-readable, portable, and automatable to accelerate drug development.

At the center of clinical trial reporting lies a category of software that has received comparatively little attention: the SAS macro library. SAS remains the dominant programming language for regulatory submissions to the United States Food and Drug Administration (FDA), with estimates suggesting that 70%–80% of electronic submissions rely on SAS-generated outputs (U.S. Food and Drug Administration, 2024; Shostak, 2014). Most large pharmaceutical companies maintain in-house macro libraries containing hundreds of SAS macros that automate the production of Tables, Figures, and Listings (TFLs) required for regulatory filings (Fu and Wu, 2004; Muller, 2014).

These libraries are substantial engineering artifacts. A typical large-pharma macro library contains 200 to 600 callable components and tens of thousands of lines of code, developed incrementally over 10–20 years (Redner and Coughlin, 2007). Each macro typically combines data preparation, statistical computation, result formatting, and output rendering within a single monolithic file. The libraries encode validated domain knowledge — correct statistical methods, regulatory-compliant formatting rules, and organization-specific conventions — that represents years of accumulated expertise (Carpenter, 2016).

However, the same architectural choices that enabled rapid growth now create serious obstacles. No machine-readable intermediate output exists between statistical computation and rendered output. AI agents cannot programmatically inspect results locked inside Rich Text Format (RTF) formatting. Cloud migration requires untangling tightly coupled dependencies. Adding a new output format requires modifying every rendering macro. And new programmers face a steep learning curve when confronting macros exceeding 2,000 lines (Bass et al., 2021).

These technical obstacles carry direct clinical and regulatory consequences. When a submission team cannot extract machine-readable results from its reporting pipeline, cross-trial pharmacovigilance becomes a manual exercise in visual comparison across hundreds of RTF tables — a process that is slow, error-prone, and resistant to the systematic signal detection that regulatory agencies increasingly expect. Bottlenecks in legacy macro maintenance slow the production of TFLs required for New Drug Application (NDA) and Biologics License Application (BLA) submissions, directly extending time-to-market for investigational therapies. Safety and efficacy data trapped in opaque formatting cannot be repurposed for meta-analyses, real-world evidence comparisons, or AI-assisted clinical review. Modernization is therefore a prerequisite for accelerating drug approvals and enabling the data-driven pharmacovigilance that modern regulatory science demands.

The pressure to modernize comes from multiple directions. Cloud platforms such as SAS Viya and Domino Data Lab require software that can operate in containerized, stateless environments (SAS Institute, 2023). LLMs are demonstrating value in clinical data management (Thirunavukarasu et al., 2023; Van et al., 2024) but require structured, machine-readable input. The Clinical Data Interchange Standards Consortium (CDISC) Analysis Results Standard (ARS) defines a metadata model for analysis results (Clinical Data Interchange Standards Consortium, 2024) but provides no migration methodology. Regulatory modernization initiatives such as ICH E8 (R1) encourage electronic workflows (International Council for Harmonisation, 2021).

Despite the urgency, the published literature offers limited guidance. PharmaSUG and SAS Global Forum proceedings describe individual macro management practices (Fu and Wu, 2004; Muller, 2014), and CDISC publications define target data models (Clinical Data Interchange Standards Consortium, 2024). To our knowledge, no work we identified presents an *integrated* methodology that combines systematic evaluation, non-destructive architectural redesign with AI integration, and rigorous cell-level parity validation in a single regulation-aware pathway (Table 1); we state our novelty at this component-combination level rather than as a claim of unqualified priority (Section 4.3). The published options share a common implicit assumption: that AI readiness requires source-level change to the legacy library. This assumption is the primary obstacle to industrial adoption, because source-level change in a regulated environment triggers re-validation costs that often exceed the perceived benefit. This raises a central question: *can a non-destructive metadata layer — one that wraps but does not modify validated SAS source — achieve AI-ready, machine-readable output from a legacy macro library while preserving regulatory standing?*

**TABLE 1:** Published approaches to SAS macro library modernization in pharmaceutical reporting.

Approach	Representative work	Scope	Evaluation	AI-ready	Regulatory	Parity
Macro management best practices	PharmaSUG (Fu and Wu, 2004; Slaughter and Carpenter, 2018; James et al., 2006)	Individual macros	No	No	Unchanged	No
Centralized autocall libraries	SAS Global Forum /PharmaSUG (Muller, 2014; Redner and Coughlin, 2007)	Library organization	No	No	Unchanged	No
R-based clinical reporting	pharmaverse /Tplyr (R Core Team, 2024; pharmaverse, 2024)	Full rewrite	No	Partial	Re-validate	Manual
Commercial compliance tools	Pinnacle 21 [32]	Compliance checking	No	No	Vendor	No
CDISC Analysis Results Standard	CDISC ARS (Clinical Data Interchange Standards Consortium, 2024)	Data model	No	Partial (model)	Standard	No
This work	Present study	Full library (558)	Yes	Yes	Traceability	Yes (365)

Eval., systematic evaluation using quantitative metrics; Reg., regulatory compliance pathway.

This paper addresses this gap by presenting a systematic framework for evaluating and modernizing legacy SAS macro libraries for AI-ready clinical trial reporting, with an explicit emphasis on a *non-destructive* adoption pathway. The framework’s central design choice is that AI readiness is achieved through a metadata layer that wraps the legacy library, rather than through modification of legacy SAS source. Source-level consolidation, where desired, is then offered as an *optional* incremental upgrade governed by the same metadata. The ultimate goal of this architecture is AI-driven pharmacoinformatics: enabling large language models and automated analytics to reason over clinical trial results through a structured Intermediate Representation (IR) that acts as the semantic bridge between validated statistical computation and machine-readable output. The framework makes six contributions:

1. A systematic evaluation methodology for legacy SAS macro libraries, comprising component taxonomy, dependency analysis, complexity metrics, and coverage assessment, applicable to any pharmaceutical organization.
2. A non-destructive adoption pattern that wraps existing SAS macros through a bridge map and IR, exposing legacy outputs as structured JSON consumable by AI, R, and Python tools without modifying validated source code, and supporting incremental consolidation as an opt-in upgrade rather than a precondition.
3. A polyglot, layered architecture organized as a five-phase pipeline supporting two deployment modes — coexistence (wrap-only) and consolidation (incremental replacement) — where YAML configuration replaces hard-coded SAS parameters and the IR decouples computation from rendering.
4. AI-cooperative interfaces built on the IR, including JSON export for large language models, R and Python interoperability through standard JSON parsers, automated Statistical Analysis Plan (SAP) parsing, and configuration generation.
5. A parity validation methodology using a bridge-map-driven harness that compares legacy and modernized outputs at the cell level, classifies divergences via a reusable error taxonomy, and operates through a gate-based verification workflow.
6. An empirical case study applying the framework to a 558-component industrial library, demonstrating non-destructive AI integration plus an optional 92% code reduction, validated on both an internal real-data study (PROT008-SR1, an internal Phase III protocol, 11/14 reports at ≥80% parity) and the public CDISC CDISCPilot01 benchmark dataset (5/5 reports at 100% regression-consistency).

The remainder of this paper is organized as follows. Section 2 reviews related work. Sections 3.1–3.3 present the three-phase methodology: evaluation, architecture design (including the non-destructive adoption pattern and the IR that enables AI integration), and migration validation. Section 4.1 applies the methodology in an industrial case study. Section 4.2 then provides the culmination of the paper: a demonstration that the IR produced by the non-destructive architecture enables AI-driven pharmacoinformatics capabilities — automated pharmacovigilance, table summarization, and configuration generation — that are structurally infeasible with legacy RTF output. Section 4.3 compares the approach with alternatives. Section 5 discusses implications, limitations, and future work, and Section 6 concludes.

## 2 Background and related work

### 2.1 Legacy SAS macro libraries in pharmaceutical reporting

SAS macro libraries are collections of parameterized SAS programs (%macro definitions) that automate TFL generation for regulatory submissions. In large pharmaceutical companies, these libraries evolve over many years as programmers add macros for new analysis types, therapeutic areas, and regulatory requirements (Fu and Wu, 2004; Muller, 2014).

Several architectural patterns recur. Each macro tends to be monolithic: a single file containing data preparation, computation, formatting, and rendering. Growth occurs through copy-and-modify, leading to near-duplicate variants. Cross-cutting concerns such as error handling and logging are reimplemented in each macro rather than centralized (Redner and Coughlin, 2007). PharmaSUG proceedings document incremental improvement approaches (Slaughter and Carpenter, 2018; James et al., 2006), but these focus on existing architectures rather than systematic redesign.

### 2.2 CDISC standards and clinical reporting

CDISC defines data standards underpinning modern clinical trial reporting. The Study Data Tabulation Model (SDTM) standardizes raw clinical data, and the Analysis Data Model (ADaM) defines analysis-ready datasets (Clinical Data Interchange Standards Consortium, 2021). More recently, CDISC introduced the Analysis Results Standard (ARS), which provides a metadata model for representing analysis results independently of visual presentation (Clinical Data Interchange Standards Consortium, 2024). ARS defines concepts such as analysis sets, groupings, methods, and result displays. In practice, however, ARS provides a conceptual data model but not an execution framework, migration methodology, or validation harness.

### 2.3 AI and automation in clinical trials

Recent LLM advances have generated interest in clinical trial AI applications, including automated literature screening (Nye et al., 2018), protocol optimization (Liu et al., 2021), adverse event detection (Salas et al., 2022), and medical writing assistance (Thirunavukarasu et al., 2023). Within statistical programming, early work explores LLM-based SAS code generation (Chen et al., 2023), automated TFL quality control (Malcolm, 2019), and LLM-based generation of clinical trial tables and figures (Yang et al., 2024).

A critical barrier to AI integration is the output format of current systems. Legacy libraries produce RTF files encoding visual formatting but not semantic content. LLMs must perform format parsing before content reasoning — a fragile process. Structured, machine-readable representations would enable reliable AI interaction, but legacy libraries provide no such output (Luo et al., 2022).

### 2.4 Software modernization in regulated industries

Modernizing legacy software under regulatory constraints is not unique to pharmaceutical programming. Banking has extensive experience migrating COBOL systems while maintaining compliance (Sneed, 2010). Aviation software governed by DO-178C has established patterns for incremental migration with continuous verification (RTCA. et al., 2012). Medical device software uses risk-based validation approaches (U.S. Food and Drug Administration, 2002). Common patterns include layered architectures isolating validated logic, intermediate representations decoupling computation from presentation, metadata-driven configuration, and automated regression testing (Comella-Dorda et al., 2000).

### 2.5 Gap analysis

Table 1 summarizes published approaches to SAS macro library modernization. To our knowledge, no published work we identified integrates, within a single methodology, (a) systematic evaluation using quantitative software engineering metrics, (b) architectural redesign preserving validated SAS logic while introducing AI-cooperative interfaces, and (c) automated parity validation providing quantitative equivalence evidence; each capability exists in isolation in prior art, and it is their combination that we present as novel (Section 4.3).

Full platform rewrites in R or Python (R Core Team, 2024; Van Rossum and Drake, 2009) abandon validated SAS logic and require complete re-validation. Commercial tools such as Pinnacle 21 (Pinnacle 21, 2024) focus on CDISC compliance checking. CDISC ARS (Clinical Data Interchange Standards Consortium, 2024) defines a target metadata model without a migration pathway. Incremental refactoring (Slaughter and Carpenter, 2018) improves existing libraries without achieving architectural separation. The framework presented here addresses all three aspects within a single methodology.

## 3 Materials and methods

### 3.1 Evaluation framework

Before redesigning a legacy SAS macro library, one must understand what it contains, how its components relate, and where complexity concentrates. This section presents a systematic evaluation methodology comprising four activities: classifying components by function, extracting dependency structure, measuring complexity, and assessing coverage. Together, these produce a quantitative profile that identifies consolidation opportunities, pinpoints high-risk components, and establishes a modernization baseline.

#### 3.1.1 Component taxonomy

A legacy library typically grows by accretion without a governing architecture. The first evaluation step classifies every callable component into one of six functional types.

Data Preparation macros subset, merge, derive, or transform ADaM datasets prior to statistical analysis. They handle tasks such as filtering treatment arms, adding total columns, deriving analysis flags, and restructuring data for specific report layouts. In mature libraries, these macros often exhibit the highest redundancy because similar transformations are reimplemented for different report types rather than parameterized once.

Statistical Computation macros invoke SAS/STAT or Base SAS procedures to produce quantitative results: descriptive statistics, frequency counts, survival estimates, regression models, shift tables, and inferential tests. These macros encode the validated analytical logic that regulatory agencies rely on and therefore require the most careful handling during modernization.

Formatting and Display macros convert raw statistical output into presentation-ready structures. They apply rounding rules, attach labels, construct display strings (e.g., “n (%)”), and arrange rows and columns according to shell templates. In legacy systems, formatting logic is often entangled with computation, making it difficult to modify output appearance without risking analytical correctness.

Rendering macros produce final output files in RTF, PDF, or other delivery formats. They manage page layout, headers, footers, column widths, and footnotes. Because rendering depends on low-level RTF or Output Delivery System (ODS) control strings, these macros tend to be fragile and resistant to reuse across different output types.

Utility and Helper macros provide shared services consumed by other macros: string manipulation, date handling, error checking, logging, parameter validation, and metadata lookup. A well-factored library concentrates these in a small utility layer; a poorly factored one scatters them as inline code.

Orchestration macros control execution flow. They read configuration, sequence macro calls, manage dataset state between pipeline stages, and handle error recovery. In legacy libraries, orchestration logic is frequently embedded in individual macros rather than separated into a distinct layer.

This taxonomy reveals where complexity concentrates and identifies consolidation targets. In the case study (Section 4.1), formatting and display accounted for the largest single category, while statistical computation represented only 22% of components, indicating that the majority of code was devoted to infrastructure rather than domain logic. When multiple macros of the same type perform nearly identical operations, they can be replaced by a single parameterized component — a pattern exploited in the redesigned architecture (Section 3.2).

#### 3.1.2 Dependency analysis

The second activity extracts the call graph: a directed graph where nodes represent macros and edges represent invocation relationships. The extraction operates in two passes: first, parsing every %macro header to identify declared names and parameter lists; second, scanning each body for call patterns (%macroname (). The scanner handles SAS-specific complications including dynamic name construction (&&varandi indirection) and conditional invocation, flagging dynamic names for manual review.

The resulting directed graph supports four diagnostic queries. Dependency clusters are groups of macros that call each other frequently, forming tightly coupled subsystems that suggest natural module boundaries. Orphan macros are declared but never called by any other macro; they may be entry points invoked from driver programs, or dead code. Circular dependencies indicate macros that call each other directly or transitively; while SAS permits this, circular calls complicate testing and increase recursion risk. Hub macros have high in-degree (called by many other macros); changes to a hub macro carry elevated regression risk and warrant prioritized test coverage.

In practice, the call graph also exposes hidden coupling. Two macros that appear independent may share state through global macro variables, temporary datasets, or naming conventions. The static graph is supplemented by scanning for %global and %sysfunc (exist ()) references to surface these implicit dependencies.

#### 3.1.3 Complexity metrics

Five metrics quantify internal complexity.

Lines of code (LOC) counts non-blank, non-comment lines. While LOC is a coarse measure, extreme values are informative: macros exceeding 500 lines almost always conflate multiple concerns and are strong candidates for decomposition.

Parameter count is extracted from the %macro header. A high count (above 20) typically signals that a macro attempts to serve too many use cases through conditional logic rather than composition.

Nesting depth measures the maximum level of %do/%end and do/end block nesting. Deep nesting (beyond four levels) correlates with control flow that is difficult to read, test, and maintain (McCabe, 1976).

Efferent coupling counts distinct external macros called by a given macro. High efferent coupling means the macro depends on many components; a change in any callee may require retesting.

Cohesion assesses whether a macro performs a single well-defined function or conflates multiple concerns. Unlike the preceding metrics, cohesion is evaluated qualitatively: high (single concern), medium (two related concerns), low (three or more concerns). Low-cohesion macros are the primary targets for refactoring into separate pipeline stages.

These metrics are collected programmatically where possible and stored in a structured inventory alongside the taxonomy and dependency data.

#### 3.1.4 Coverage assessment

The fourth activity maps the library against reporting requirements. In the pharmaceutical context, clinical study reports follow structures defined by ICH E3 (International Council for Harmonisation, 1995) and use datasets conforming to CDISC ADaM (Clinical Data Interchange Standards Consortium, 2021). A typical submission includes demographic tables, adverse event summaries, laboratory shift tables, efficacy analyses, and time-to-event displays.

The assessment proceeds in three steps. First, each macro is mapped to the report type or types it supports — a mapping that is often implicit in legacy libraries and must be verified by inspecting code. Second, the mapping is analyzed for functional overlap: cases where multiple macros produce structurally similar outputs, indicating redundancy from study-specific copying. Third, the mapping is checked for gaps: report types not covered or only partially covered. The ratio of unique analytical functions to total components quantifies redundancy; a low ratio confirms that consolidation can reduce the library’s footprint by half or more without sacrificing functional breadth.

#### 3.1.5 Evaluation summary

The four activities produce a structured profile: a classified component inventory (Table 2, legacy column), an annotated dependency graph, a complexity distribution with outliers flagged, and a coverage matrix. Figure 1 presents the taxonomy breakdown for the case-study library. The methodology requires no proprietary tools and is applicable to any pharmaceutical SAS macro library.

**TABLE 2:** Software engineering metrics: legacy library vs. modernized framework.

Metric	Legacy library	Modernized framework	Change
Total callable components	558	158 SAS files	− 72%
Total SAS lines of code	372,698	28,340	− 92%
Avg. lines per SAS file	932	179	− 81%
YAML configuration files	0	147 (39 report types)	0 → 147
Python modules	0	48 (15,855 LOC)	0 → 48
Total framework LOC	372,698 (SAS only)	∼ 63,000 (SAS + Python + YAML)	− 83%
Machine-readable IR output	No	Yes (JSON export)	Yes
Render format support	RTF only	RTF, PDF, HTML, JSON	+3 formats
Bridge map entries	N/A	365 (full invocation coverage)	Full coverage
Centralized report registry	No	39 report types	0 → 39
Audit trail /execution manifest	Manual	Automated (fw_audit)	Automated
AI-cooperative interfaces	None	SAP parser + config gen + IR JSON	Yes

**FIGURE 1:**
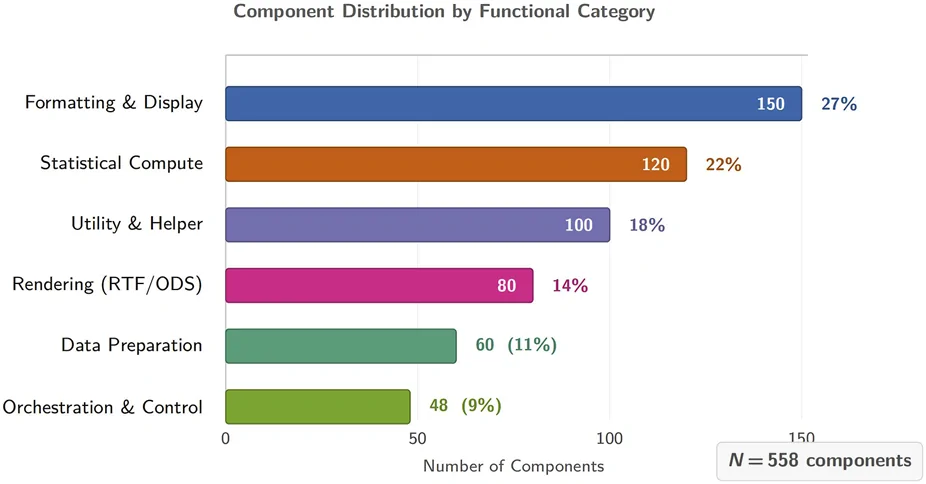
Component taxonomy of the legacy 558-macro library, classified by functional role. Formatting and display macros constitute the largest category (27%), reflecting the high proportion of code dedicated to output appearance rather than statistical computation.

### 3.2 Architecture design

The framework architecture addresses the structural deficiencies identified in Section 3.1 through a layered, metadata-driven pipeline with explicit contracts at every phase boundary. This section presents the design principles, pipeline structure, IR, configuration model, AI-cooperative interfaces, regulatory-traceability design features, and the non-destructive adoption pattern that allows the architecture to be deployed without modifying legacy SAS source.

#### 3.2.1 Design principles

Five principles guide the architecture:

##### 3.2.1.1 Separation of concerns

Each macro performs exactly one function and declares its inputs and outputs through a standard interface, eliminating the monolithic macros identified in the legacy evaluation.

##### 3.2.1.2 Metadata-driven configuration

YAML configuration files replace hardcoded parameters. Report definitions, population filters, treatment arm labels, and pipeline sequences are specified declaratively. Modifying a report requires editing YAML, not SAS code (Fowler, 2002).

##### 3.2.1.3 Format-agnostic intermediate representation

An IR sits between computation and rendering. The compute layer produces results in a standard schema; the render layer reads a typed cell grid and emits RTF, PDF, HTML, or JSON. Adding a new output format requires only a new render engine.

##### 3.2.1.4 AI-cooperative interfaces

All pipeline artifacts — configurations, IRs, execution manifests — are available in structured formats (YAML, JSON), enabling LLMs and AI agents to read results, generate configurations, and participate in quality control without parsing proprietary formats.

##### 3.2.1.5 Regulatory traceability by design

Audit trails, execution manifests, input/output hashing, and electronic-signature hooks are architectural requirements. Every pipeline step records provenance metadata sufficient to reconstruct how a given output was produced. These are *design features* intended to support regulatory traceability, not a claim of validated compliance; no formal IQ/OQ/PQ qualification has been performed (Section 5).

#### 3.2.2 Five-phase pipeline

The framework organizes clinical report production into five sequential phases with explicit input and output contracts (Figure 2).

**FIGURE 2:**
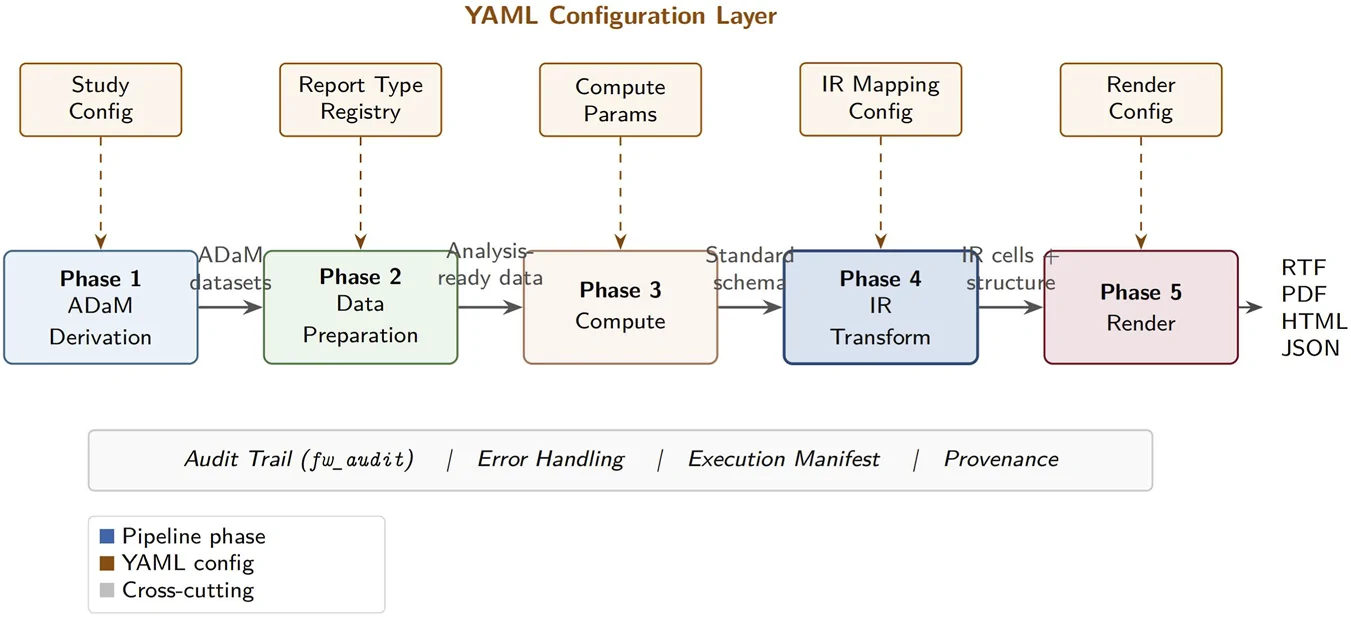
Five-phase pipeline architecture with explicit input/output contracts at each layer boundary. YAML configuration (top) feeds into every phase, while the Intermediate Representation between Compute and Render serves as the key decoupling contract. Cross-cutting concerns span all phases.

##### 3.2.2.1 Phase 1: ADaM derivation

This optional phase generates ADaM datasets from SDTM source data using YAML-configured derivation rules. Seven macros handle major ADaM structures: ADSL, BDS, OCCDS, and ADTTE, plus generic derivation, safety algorithm, and validation modules.

##### 3.2.2.2 Phase 2: data preparation

Data preparation macros transform ADaM datasets into analysis-ready form: subsetting, derivation, flag creation, ADSL merging, row completion, treatment sorting, and total column addition. Every macro follows a standard interface (input_ds, output_ds, operation-specific parameters, execution_id, debug). The pipeline sequence for a given report type is specified in YAML.

##### 3.2.2.3 Phase 3: compute

Compute macros execute statistical analyses on prepared data, covering descriptive statistics, frequency counts, incidence rates, Kaplan-Meier estimation, log-rank tests, chi-square tests, shift tables, change from baseline, ANOVA, regression, mixed models, and subgroup analyses. Every compute macro produces output in a standard schema containing grouping variables, treatment identifiers, statistic names, numeric values, formatted strings, and method identifiers. This standard schema is the critical contract enabling downstream phases to operate uniformly.

##### 3.2.2.4 Phase 4: IR transform

Three components convert compute output into the IR (described in Section 3.2.3): cl_to_ir performs the transformation, cl_ir_reconcile verifies numeric integrity by tracing cells back to source statistics within configurable tolerance (default $1\times 10^{-10}$ ), and fw_validate_ir checks structural constraints.

##### 3.2.2.5 Phase 5: render

Render engines read IR datasets and produce final output. SAS engines generate RTF for regulatory submission; Python engines generate JSON for AI consumption, HTML for interactive review, and PDF for archival. Render engines contain no statistical logic — they are pure format translators.

Each phase boundary enforces an explicit contract: the output schema of phase N is the required input of phase N+1 , enabling independent testing, replacement, or extension of any phase (Figure 3).

**FIGURE 3:**
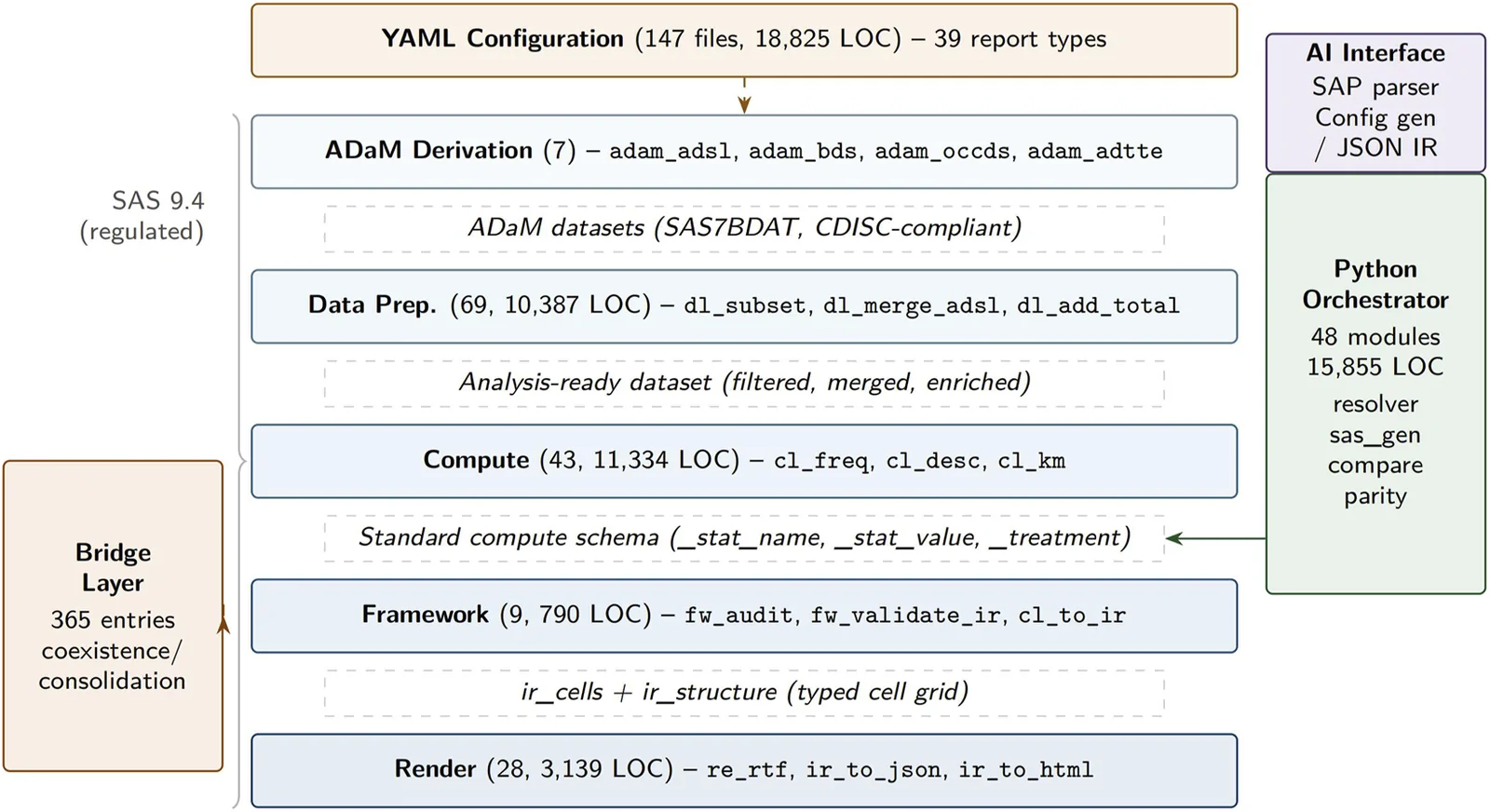
Layered architecture showing six SAS layers (ADaM Derivation, Data Preparation, Compute, Framework, Bridge Compatibility, Render) with explicit contracts at each boundary, plus the Python orchestration layer and AI interfaces. The bridge layer provides a 365-entry facade over the legacy library.

#### 3.2.3 Intermediate representation

The IR is the central architectural innovation, representing a clinical report as two complementary datasets (Figure 4).

**FIGURE 4:**
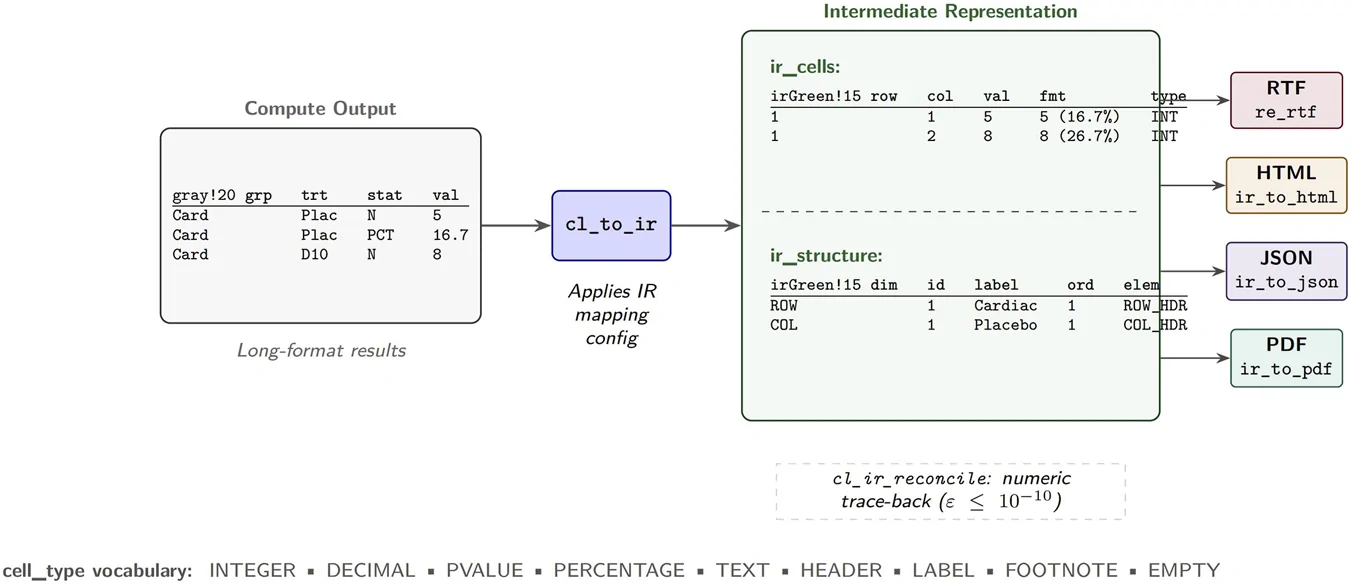
Intermediate Representation (IR) schema showing the decoupling of statistical computation from rendering. The cl_to_ir macro transforms long-format compute output (left) into a grid-based cell model consisting of ir_cells and ir_structure datasets (centre). The format-agnostic IR feeds multiple render engines (right). Numeric integrity is verified by cl_ir_reconcile.

ir_cells contains one row per cell in the report grid: row_id, col_id, raw numeric cell_value, formatted display string cell_formatted, and cell_type from a controlled vocabulary (INTEGER, DECIMAL, PVALUE, PERCENTAGE, TEXT, HEADER, LABEL, FOOTNOTE, EMPTY). Numeric types carry both raw values and formatted strings; non-numeric types carry only display content. This controlled vocabulary is the key enabler of AI-driven pharmacoinformatics (Section 4.2): by semantically classifying each cell, the vocabulary allows downstream consumers — including large language models — to distinguish a p-value from a percentage, a column header from a data row, and a statistical result from a footnote, without heuristic parsing of rendered output. Every cell also carries report_id, execution_id, and sort_order.

ir_structure defines the row and column dimensions: dimension (ROW or COL), dim_id linking to ir_cells, display label, sort order, indent level, text alignment, span count, and element_type (COLUMN_HEADER, ROW_HEADER, DATA_ROW, TOTAL_ROW, SEPARATOR, SPANNING_HEADER).

Four structural rules govern IR validity: completeness (every cell pair has corresponding structure definitions), contiguity (identifiers start at 1 with no gaps), uniqueness (each grid position appears once), and consistency (report_id and execution_id match across datasets).

This design enables cell-level QC on structured data rather than rendered text, format-agnostic output from a single IR, and reconciliation against source compute output.

#### 3.2.4 YAML registry and configuration

The configuration model has three tiers. The report type registry contains one YAML file per report type, defining pipeline steps, parameters, IR mapping rules, and render configuration. The registry currently defines 39 report types. Study-level configuration provides per-study overrides: populations, treatment labels, dataset locations. Parameter resolution occurs through a Python bridge (yaml_to_sas) that reads YAML, resolves cross-references, and emits SAS macro variables, handling type conversion and the SAS 32-character name limit.

#### 3.2.5 AI-cooperative interfaces

The architecture provides three AI integration points. JSON export (ir_to_json) exports IR datasets as structured JSON with typed columns and cell arrays, enabling LLMs to reason about clinical results as structured data. SAP document parser (sap_parser) extracts table specifications from SAP documents. Configuration generator (config_generator) produces draft YAML configurations from parsed SAP output, with a matcher module mapping specifications to registry entries. Generated configurations require human review — the framework inserts an explicit review gate.

The design choice is that AI agents interact with IR and YAML layers, not raw SAS output. Structured data enables structured reasoning: an LLM can identify that a p-value cell contains 0.0312 and the method is chi-square, without parsing RTF layout. Throughout this paper, *AI readiness* is used operationally: it denotes the property that every compute step’s output is available as typed JSON conforming to the IR schema, consumable by LLMs, R, and Python without format-specific parsing.

#### 3.2.6 Regulatory-traceability design features

##### 3.2.6.1 Audit trail

The fw_audit macro records every pipeline step with timestamp, user identifier, event type, step name, status, layer, and comments. Records accumulate in a persistent SAS dataset.

##### 3.2.6.2 Execution manifest

Each pipeline run records input datasets with file hashes, resolved parameters, macro versions, SAS session metadata, and output hashes — sufficient to answer “what exact inputs and parameters produced this output?”

##### 3.2.6.3 Electronic signature support

Audit trail and manifest structures are designed to support 21 CFR Part 11 traceability principles (U.S. Food and Drug Administration, 1997) — including user authentication linkage and tamper-evident storage — pending formal qualification; the framework does not itself assert Part 11 compliance.

##### 3.2.6.4 Change control

YAML report definitions in version control produce reviewable, diff-able commits readable by both programmers and non-programmer reviewers.

The combination of audit trail, execution manifest, and version-controlled configuration creates a traceability chain from submission output through every transformation step to source ADaM datasets and SAP specification (Cleland-Huang et al., 2012).

#### 3.2.7 Non-destructive adoption pattern

The bridge map is the single artifact that distinguishes the two deployment modes. The Native Driver does not need to know which mode applies to any given entry: it simply executes the entry’s native_target. The deployment surface that an organization manipulates over time is therefore the metadata, not the SAS source. This indirection has three consequences. First, the regulatory standing of the legacy macros is preserved by default, because their files are read but never written by the framework. Second, the rate of consolidation is a business decision rather than an architectural commitment — an organization may consolidate aggressively, conservatively, or not at all, using the same framework. Third, AI readiness is decoupled from consolidation: the IR and JSON export are produced by the metadata layer regardless of whether the underlying computation originated in a legacy macro running in coexistence mode or in a modern core macro running in consolidation mode. A pharmaceutical organization can therefore integrate AI workflows on day one without committing to any source-level modernization.

##### 3.2.7.1 Polyglot integration

The non-destructive adoption pattern is what makes polyglot integration practical in a regulated setting. SAS retains exclusive responsibility for regulated computation, including in coexistence mode where the original macros run unchanged within their existing validation envelope. Python operates outside the regulated boundary, providing pipeline orchestration, IR-to-JSON serialization, the SAP parser, and the parity validation harness. R consumes the JSON IR through standard parsers (e.g., jsonlite) for exploratory analytics, custom visualizations, and statistical cross-checks, without re-implementing the validated SAS computation. Large language models receive the same JSON IR and operate on its typed cell structure, eliminating the brittle RTF parsing that has prevented LLM integration in legacy workflows. Each language addresses a single concern, the IR is the single integration surface, and the legacy library remains the authoritative source of regulated computational logic. This division of labor is consistent with the recommendations of Good Automated Manufacturing Practice (GAMP) 5 (International Society for Pharmaceutical Engineering, 2022) for computerized systems in regulated industries, which advocates strict separation between validated computational components and unvalidated supporting infrastructure, and with broader software-engineering guidance on legacy system migration in safety-critical and regulated domains (Comella-Dorda et al., 2000).

##### 3.2.7.2 Implications for adoption risk

The non-destructive pattern materially changes the adoption-risk profile of clinical reporting modernization. Under conventional rewrite approaches, an organization commits up-front to a multi-year project whose value is realized only on completion. Under the coexistence-first pattern proposed here, AI readiness is available at initial deployment — obtained by populating the bridge map and standing up the metadata layer — rather than only on completion of a multi-year rewrite; consolidation, where elected, becomes a continuous improvement activity whose individual increments can be evaluated, validated, and accepted independently. This staged value delivery aligns with risk-based validation frameworks such as GAMP 5 (International Society for Pharmaceutical Engineering, 2022) and reduces the financial and regulatory commitment required to begin modernization.

#### 3.2.8 Non-destructive IR extraction mechanism

A recurring question is how the framework obtains a typed IR from an unchanged legacy macro whose only documented output is opaque RTF. The mechanism does *not* parse RTF. Instead, each bridge-map entry carries adapter metadata that identifies the macro’s *tabular output boundary* — the point at which the macro has already materialized its results as a SAS dataset, before those results are rendered to RTF. The orchestrator captures that dataset and applies a typed-cell transform to produce ir_cells and ir_structure (Section 3.2.3).

Three capture archetypes (Gamma et al., 1994) cover the legacy macros encountered in the case study. ODS OUTPUT capture intercepts the output object of a SAS procedure (e.g., PROC FREQ or PROC MEANS) via an ODS OUTPUT statement injected by the adapter preamble, leaving the macro body unchanged. Emitted-dataset capture reads a work dataset the macro already creates as a side effect of computation. Upstream-dataset capture reads the analysis dataset immediately before the macro’s rendering step. In all three cases the legacy computation executes verbatim; only the *observation point* is added.

The adapter metadata fields are: native_target (the legacy or modern-core macro to invoke), output_dataset (the dataset to capture), column_role_map (which columns are row labels, treatment groups, or statistics), cell_type_map (how raw columns map to the controlled cell_type vocabulary), and sort_key (the ordering that fixes sort_order). Because capture operates on typed SAS datasets rather than rendered text, the RTF-specific hazards of a parsing approach — merged cells, footnote runs, Unicode fallbacks, indentation, spanning headers, and multi-level treatment columns — do not arise at capture time; they are represented downstream as structural attributes of the IR (element_type, span count, indent level) rather than as text to be recovered.

##### 3.2.8.1 Minimal worked example

Listing 1 traces one demographics row from a single legacy macro call to IR cells and exported JSON.

Listing 1 Minimal end-to-end example: one legacy macro call, its bridge-map entry, the resulting IR cell, and the exported JSON.

/* 1. Legacy call (source unchanged) */
%demographics(ds=adsl, trt=TRT01A, out=work.t1412);
# 2. Bridge-map entry (YAML)
 - legacy_id: demographics_t1412
  native_target: demographics       # coexistence: legacy macro
  output_dataset: work.t1412
  column_role_map: {ROWLBL: row_header, TRT01A: group, COL1: value}
  cell_type_map: {COL1: PERCENTAGE}
  sort_key: [ROWLBL]
/* 3. Captured IR cell (ir_cells, one row shown) */
report_id=T-14.1.1 row_id=4 col_id=2
cell_value=61.6 cell_formatted=“53 (61.6%)” cell_type=PERCENTAGE
// 4. Exported JSON (ir_to_json)
{“report_id”:“T-14.1.1”,”row_id”:4,”col_id”:2,
 “cell_value”:61.6,“cell_formatted”:“53 (61.6%)”,“cell_type”:“PERCENTAGE”}

### 3.3 Migration and parity validation

Modernizing a legacy library is insufficient unless the new framework produces equivalent outputs. This section presents the parity validation methodology: executing legacy and modernized pipelines on identical input data, comparing outputs at the cell level, and triaging discrepancies through a gate-based workflow.

#### 3.3.1 Bridge map

The central artifact is the *bridge map*: a structured registry establishing one-to-one correspondence between each legacy macro invocation and its modernized counterpart (Evans, 2003). Each entry contains:

- legacy_id: Unique identifier for the legacy macro call (e.g., ae_summary_soc).
- native_target: The modernized pipeline configuration replacing the legacy call.
- parameter_mapping: Explicit mapping from legacy to modern parameter names, accommodating renames and semantic shifts.
- defaults: Values for parameters existing only in the new framework, capturing design decisions implicit in legacy code.
- preamble_sas: Optional SAS code establishing preconditions before the native pipeline call.
- post_calls: Optional follow-up processing steps.

The bridge map is defined in machine-readable format (YAML or JSON) for programmatic consumption. It documents every migration decision in a single auditable location and enables automated construction of paired execution plans.

#### 3.3.2 Parity harness

The parity harness is a dual-driver execution engine that orchestrates side-by-side comparison of legacy and modernized outputs.

The LegacyDriver accepts a bridge map entry and constructs a SAS program that invokes the original macro with its original parameters on a specified input dataset. It captures the resulting output files (typically RTF), execution logs, and timing data.

The NativeDriver accepts the same bridge map entry and constructs the corresponding modernized pipeline invocation, translating parameters through the bridge map’s parameter_mapping and defaults fields, prepending any preamble_sas, and appending any post_calls. It captures the same artifact types as the LegacyDriver.

Both drivers operate on identical input data loaded from a common source. The harness reads the bridge map and a study-level configuration file that specifies dataset locations, library paths, and environment settings. For each bridge map entry, it executes both drivers, stores their outputs in a structured directory hierarchy organized by report type, and passes the paired outputs to the comparison engine. This dual-driver architecture isolates comparison logic from execution concerns, allowing batch mode across an entire bridge map or selective execution during iterative debugging.

#### 3.3.3 Validation strategy

The comparison engine applies type-specific validation strategies to paired outputs.

##### 3.3.3.1 Cell-level comparison for tables

The engine parses both RTF files, extracts each cell’s textual content, and performs cell-by-cell comparison. Text cells require exact string match after normalization (whitespace trimming, case folding where appropriate). Numeric cells allow an epsilon tolerance to accommodate rounding differences. This approach detects content-level discrepancies that visual inspection would miss.

##### 3.3.3.2 Structural comparison for listings

Listings are validated by comparing row count, column count, and content across corresponding cells. Because listings are typically long-form patient-level data, structural checks confirm both pipelines select the same records in the same order.

##### 3.3.3.3 Visual comparison for figures

Figures (e.g., Kaplan-Meier plots) are compared by file size as a coarse structural check, supplemented by manual spot-checking. Full pixel-level comparison is deferred as an optional extension.

Each comparison produces a verdict: PASS (all cells match within tolerance), FAIL (content divergence), ERROR (execution failure), or SKIP (excluded by explicit annotation). Results are aggregated into a summary matrix providing at-a-glance parity status.

#### 3.3.4 Gate-based verification

The methodology defines a gate-based workflow progressing from structural checks to full parity runs (Figure 5):

**FIGURE 5:**
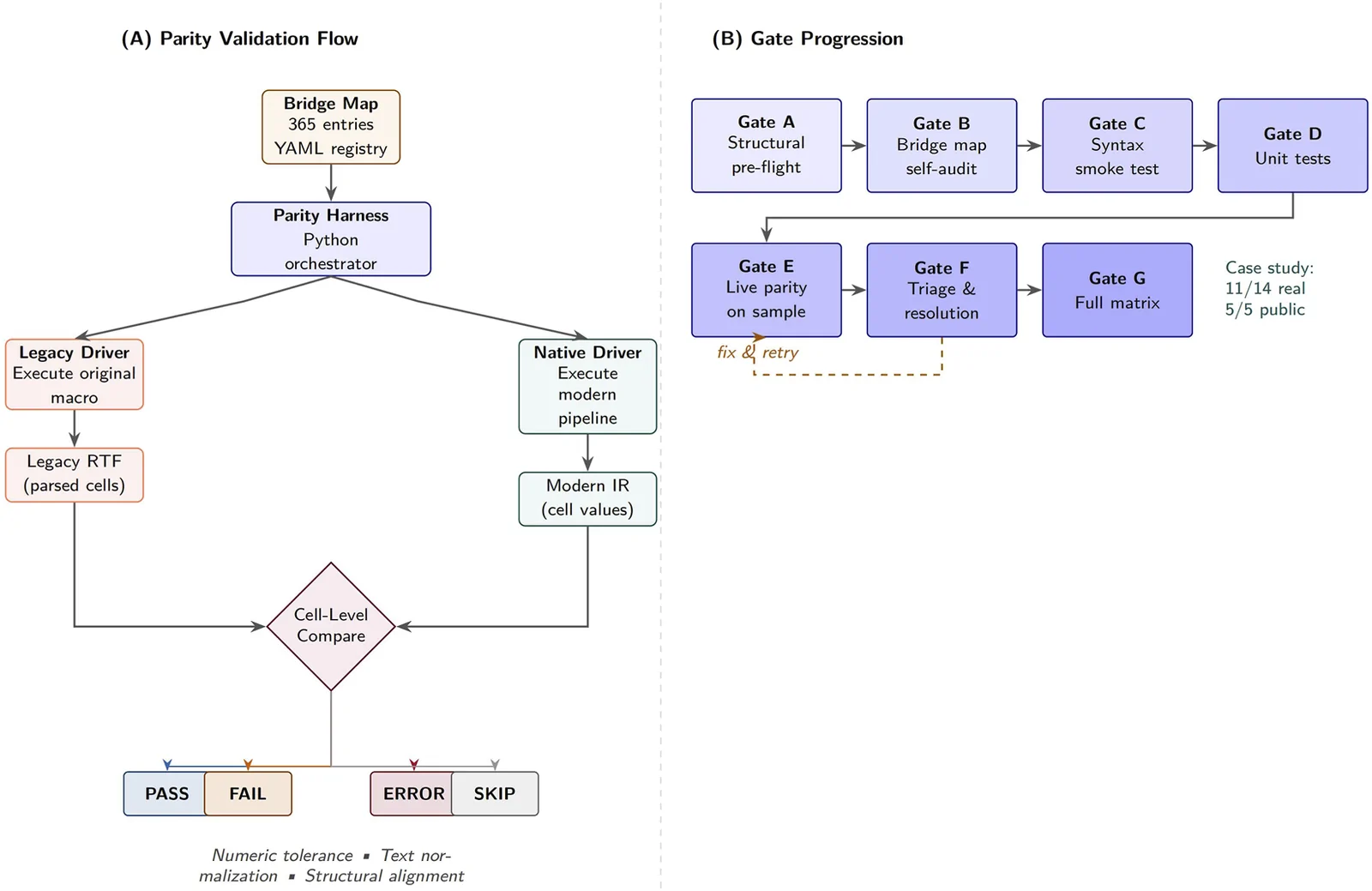
Parity validation methodology. **(A)** The parity harness executes each report through both the legacy macro library and the modernized pipeline, then performs cell-level comparison to produce a PASS/FAIL/ERROR/SKIP triage. **(B)** The seven-gate progression model ensures incremental validation from configuration syntax checks through full regression matrix.

##### 3.3.4.1 Gate A: structural pre-flight

Verify execution environment: libraries accessible, datasets exist, configurations parse.

##### 3.3.4.2 Gate B: bridge map self-audit

Validate bridge map entries: referenced macros and targets exist, parameter mappings are valid, required fields populated.

##### 3.3.4.3 Gate C: SAS syntax smoke test

Submit generated SAS programs in syntax-check mode to verify balanced macro calls and resolved variables.

##### 3.3.4.4 Gate D: unit tests

Execute unit tests for harness components and migration tools. Together, Gates C and D establish the technical preconditions for the live parity runs that follow; the 80% cell-level parity threshold used to evaluate Gate E outcomes (Section 4.1.4) is meaningful only after these structural checks confirm that both pipelines execute without error.

##### 3.3.4.5 Gate E: live parity on sample reports

Select a representative subset spanning different report types and execute full dual-driver comparison.

##### 3.3.4.6 Gate F: triage and resolution

Classify failures using an error taxonomy:

- *Infrastructure errors*: Missing libraries, incorrect paths.
- *Parameter errors*: Incorrect variable names, mismatched dataset references.
- *Semantic errors*: Different computational logic (e.g., denominator definitions).
- *Content errors*: Numerical divergence within structure-matching outputs.

Each class implies a different resolution strategy.

##### 3.3.4.7 Gate G: full matrix

After resolving issues from Gates E–F, execute the harness against all bridge map entries.

The gate sequence ensures each gate’s preconditions are established by preceding gates. Organizations can repeat Gates E–G iteratively as they expand coverage.

#### 3.3.5 Parity threshold definition and rationale

The 80% cell-level parity value used throughout the case study (Section 4.1.4) is a *triage-admission gate*, not a regulatory-equivalence criterion. A report reaching 80% cell-level agreement is admitted into the gate-based verification workflow (Figure 5), where the residual disagreement is triaged by root cause and either resolved at the framework layer or documented as a known structural feature. We adopt this gate for three reasons. First, it separates reports whose residual is dominated by a small number of systematic, framework-level causes — addressable by the fix-once /apply-many pattern of Section 4.1.4 — from reports requiring per-report investigation. Second, it provides a stable, pre-registered admission criterion for the iterative development process, so that progress across development phases is measured against a fixed bar. Third, it reflects the empirical observation that, above this level, the remaining divergence in our case study was overwhelmingly presentational (table-layout geometry, label drift) rather than computational.

We state explicitly what the 80% gate is *not*. It is not an assertion that an 80%-parity report is fit for regulatory submission. Regulatory TFLs require essentially complete agreement between legacy and modernized outputs, and layout and labeling are themselves part of a regulated deliverable. Submission-grade deployment of any individual report therefore requires the residual to be either resolved to full parity or formally justified and signed off on a per-report basis; the gate governs *entry* into that per-report process, not its outcome. This distinction is revisited in the limitations (Section 5).

## 4 Results

### 4.1 Case study

This section applies the evaluation, architecture, and validation methodologies from Sections 3.1–3.3 to an industrial SAS macro library. The case study provides quantitative evidence for the framework’s effectiveness and reports parity validation results against the public CDISC CDISCPilot01 benchmark dataset.

#### 4.1.1 Legacy library profile

The legacy library was developed incrementally over approximately 15 years on SAS 9.4. It served as the sole production system for generating TFLs across regulatory submissions in multiple therapeutic areas. At evaluation, the library comprised 558 callable macro components across 400 SAS source files, totaling 372,698 lines of code.

Applying the component taxonomy (Section 3.1) yielded the breakdown in Figure 1: formatting and display ( ∼ 150, 27%), statistical compute ( ∼ 120, 22%), utility and helper ( ∼ 100, 18%), rendering ( ∼ 80, 14%), data preparation ( ∼ 60, 11%), and orchestration ( ∼ 48, 9%). Two observations emerged. First, the largest category was formatting and display — macros encoding visual presentation logic tightly coupled to RTF. Second, statistical compute macros represented only 22% of components, indicating that most code served infrastructure rather than domain logic.

Dependency analysis revealed a deeply interconnected call graph averaging 4.7 callees per macro, with orchestration macros exhibiting fan-out exceeding 20. Circular dependencies were identified in two subsystems. No machine-readable dependency manifest existed.

Complexity metrics confirmed structural challenges: average file length 932 lines, longest files exceeding 3,000 lines. Output was exclusively RTF. Configuration was entirely through macro parameters with hard-coded defaults. No intermediate representation existed between computation and rendered output.

#### 4.1.2 Modernized framework profile

The modernized framework follows the layered architecture of Section 3.2, organizing functionality into six SAS layers (ADaM derivation, data preparation, computation, framework services, bridge compatibility, rendering), supported by Python orchestration and AI agent interface layers. Consistent with the adoption pattern of Section 3.2.7, the legacy 558-component library is retained intact as the source of regulated computation; the framework reads but does not modify any legacy macro file.

The bridge compatibility layer is the primary surface that connects the legacy library to the modern pipeline. It comprises a 365-entry bridge map providing complete invocation-level coverage of the 558 callable components in the legacy library (near-duplicate components may share one parameterized bridge entry; Table 3), plus facade wrappers that allow legacy calling conventions to resolve under both deployment modes. In coexistence mode, a bridge map entry’s native_target field references the existing legacy macro together with adapter metadata that maps its outputs onto the standard compute schema; the legacy macro executes unchanged and its output is forwarded to the IR transform phase. In consolidation mode, the same entry’s native_target field instead references a parameterized core macro from the modern compute layer, configured by YAML.

**TABLE 3:** Definitions of the software-metric units used in this section, reconciling the counts reported for the legacy library and the modernized framework.

Term	Definition
Callable component	A legacy %macro invocable by name from a driver program or another macro. The legacy library contains 558 callable components
SAS source file	A physical.sas file; one file may define several callable components. The legacy library spans 400 source files; the modern core spans 158 files
Core macro	A single-responsibility macro in the modernized compute, data-preparation, or render layer. The three core layers span 140 of the 158 modern SAS files (69 data-preparation, 43 compute, 28 render; Section 4.1)
Supporting file	A modern SAS file outside the three core layers: framework-service (9), ADaM-derivation (7), or bridge-facade (2) file — 18 files in total; 140 core-layer files plus 18 supporting files equal the 158 modern SAS files
Bridge entry	One row of the bridge map establishing a legacy-to-modern correspondence (365 entries). A single bridge entry may cover several near-duplicate callable components consolidated under one parameterized target, so 365 entries provide complete *invocation-level* coverage of the 558 callable components without being numerically equal to them
LOC	Non-blank, non-comment lines of code, counted per language (SAS, Python, YAML) and never summed across languages without labeling

For the consolidation portion of the case study, the modern SAS core comprises 158 files totaling 28,340 lines of code. The data preparation layer provides 69 macros (10,387 LOC); the compute layer provides 43 macros (11,334 LOC); the render layer provides 28 macros (3,139 LOC); the framework layer provides 9 macros (790 LOC) for logging, error handling, and audit trails; the ADaM derivation layer provides 7 macros; and the bridge compatibility layer provides 2 facade files (together accounting for the remaining ∼ 2,690 LOC).

Configuration is externalized into 147 YAML files (18,825 LOC) organized into a registry covering 39 report types, with per-study overrides for both the internal study (20 report configs) and the public CDISCPilot01 benchmark (5 report configs). The Python layer comprises 48 modules (15,855 LOC) for pipeline coordination, YAML resolution, SAS code generation, IR export, SAP parsing, and parity comparison. The orchestrator is mode-agnostic: it dispatches each report to the native_target resolved from the bridge map without needing to know whether the underlying computation originated in a legacy macro or a modern core macro. The total framework comprises 353 files and approximately 63,000 lines of code across three languages.

#### 4.1.3 Quantitative comparison

Table 2 presents the software engineering metrics for the case study, separated into two columns: the legacy library as encountered (which the framework preserves under coexistence mode) and the modern core that is reached *if and when* the organization elects to consolidate. Figure 6 illustrates the before-and-after comparison across key dimensions. We report consolidation outcomes here because they were measured in the case study, but the framework does not require consolidation; the metrics in this section therefore describe the upper-bound effect of opting in, not a precondition for AI readiness.

**FIGURE 6:**
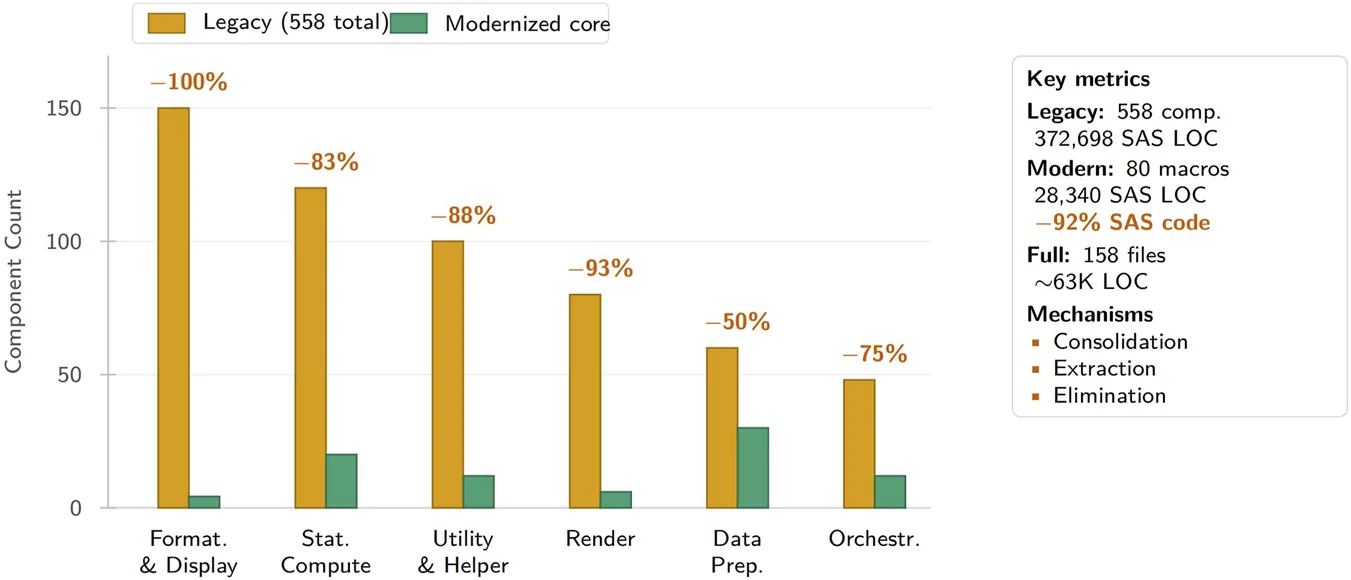
Component count reduction from legacy library (558 components) to the modernized SAS core, by functional category. The category counts after consolidation (summing to 80 category-level macros, with Formatting/Display fully absorbed) are a different unit of measurement from the architectural-layer file counts of Section 4.1 (140 core-layer +18 supporting = 158 files; Table 3); the two must not be added or compared directly. The largest reductions occur in Formatting/Display (100%, absorbed into the IR and render layer) and Statistical Compute (83%, consolidated from 120 specialized macros into parameterized core macros). Right panel shows the three reduction mechanisms and the full 158-file breakdown.

When consolidation is applied to the case-study library, the modern core comprises 158 SAS files against 558 legacy components — a 72% reduction in file count and a 92% reduction in SAS lines of code (28,340 vs. 372,698), reflecting consolidation of redundant variants, extraction of configuration into YAML, and elimination of dead code (detailed in Section 4.1.5). None of these activities is required to obtain AI-ready output; coexistence mode achieves AI readiness with the legacy component count unchanged.

Modernized macros are individually smaller (179 vs. 932 lines average), a consequence of single-responsibility design and configuration externalization. The compute layer averages 264 lines per macro (vs. legacy >500 ), with the data preparation layer at 151 lines per macro.

Beyond consolidation, the framework introduced capabilities that apply equally under both deployment modes: 147 YAML configuration files (18,825 LOC), 48 Python orchestration modules (15,855 LOC) for CI/CD and AI integration, a typed IR with JSON export consumable by LLMs and by R analytics through standard JSON parsers, and multi-format output support (RTF, PDF, HTML, JSON). The total framework spans approximately 63,000 lines across three languages. These capabilities are reached on day one of coexistence-mode deployment; they do not require any of the consolidation reductions described above.

Because the case study reports several overlapping counts, Table 3 first defines each unit of measurement; every figure quoted in this section traces to one of these definitions.

#### 4.1.4 Parity validation results

Parity validation was conducted across two complementary tracks: an internal real-data track using the PROT008-SR1 study (an anonymized industry Phase III dataset with full ADaM coverage; only aggregate parity metrics are reported in this paper, and publication permission for those derived metrics is on file with the sponsor), and a public benchmark track using the CDISC CDISCPilot01 synthetic dataset (three treatment arms; Placebo, N=86 ; Xanomeline Low Dose, N=84 ; Xanomeline High Dose, N=84 ). The two tracks serve distinct purposes. The real-data track exercises the framework against schema-aligned production inputs and provides production-velocity feedback. The public track exercises reproducibility and provides an externally verifiable benchmark for the methodology paper. While 19 of the 365 bridge map entries were subjected to full end-to-end parity validation, these 19 constitute a purposive sample covering all major structural archetypes of clinical TFLs (e.g., disposition, demographics, adverse events, laboratory shifts, time-to-event). The remaining 346 entries were validated at the unit level through gate-based structural checks (Gates A–D), which verify bridge map integrity, configuration schema compliance, IR field completeness, and render-layer coverage for each entry.

Table 4 makes the validation-coverage structure explicit, so that no claim implies full end-to-end parity across all 365 entries.

**TABLE 4:** Validation coverage by tier. Full end-to-end cell-level parity was established for 19 of 365 bridge entries; the remaining entries were validated at the mapping and unit tiers and are available in coexistence mode.

Tier	Validation activity	Entries
T1	Documented legacy-to-modern mapping (bridge entry present and self-audited)	365 /365
T2	Unit-level structural checks (Gates A–D: schema, IR field completeness, render coverage)	365 /365
T3	Coexistence-mode availability (legacy macro executes unchanged; IR captured)	365 /365
T4	Full end-to-end cell-level parity (14 internal +5 public reports)	19 /365

##### 4.1.4.1 Real-data track (PROT008-SR1)

Fourteen report types were selected for parity validation, spanning the full range of clinical TFL categories: adverse event summaries (5 reports), baseline characteristics (2), disposition, compliance, ECG summaries (2), laboratory analyses, adverse event listings, and risk management tables. Each was backed by a long-standing legacy macro that has produced regulatory submission output. Table 5 summarizes the cell-level parity results.

**TABLE 5:** Cell-level parity results on the PROT008-SR1 real-data track (14 report types). Cell counts are matched/total compared cells from the final execution artifacts of each report type.

Report type	Category	Cells (matched/total)	Parity
AE-SUMMARY	AE frequency	116 /117	**99.2%**
AE-SPECIFIC	AE frequency	539 /572	**94.2%**
BASELINE-CHAR	Descriptive statistics	507 /546	**92.9%**
AE-SPECIFIC-5	AE frequency	240 /260	**92.3%**
ECG-QTCF	Threshold counts	66 /72	**91.7%**
COMPLIANCE	Frequency	130 /143	**90.9%**
DISPOSITION	Frequency	163 /182	**89.6%**
AE-LISTING	Data listing	—^†^	**87.5%**
AE-REL	AE frequency	146 /169	**86.4%**
RMP-AE	Intensity breakdown	1,407 /1,722	**81.7%**
BASELINE-DISEASE	Descriptive statistics	581 /715	**81.3%**
AE-ANALYSIS	AE with CI/p-value	120 /184	65.2%*
ECG-SUMMARY	Descriptive wide	58 /96	60.4%*
LAB-PDLC	Threshold with CI	471 /1,050	44.9%*

* Structural ceiling: legacy uses a fundamentally different table layout orientation (treatments-as-rows or multi-sub-column transposition). The native output is statistically correct; the difference is in layout geometry only.

^†^ AE-LISTING is a patient-level listing validated by structural comparison (record counts, column counts, and ordering); cell counts are not defined for listings

Eleven of 14 reports cleared the 80% cell-level parity threshold (mean across all 14 report types 82.7%, median among the 11 clearing reports 89.6%), satisfying the predefined acceptance criterion ( ≥11/14 at ≥80% ). The iterative improvement from initial execution (8/14 at ≥80% ) to final results (11/14) was achieved through 72 targeted fixes applied across 6 development phases, all at the framework layer. The high concordance rates across the 11 passing reports — with 6 exceeding 90% cell-level parity — indicate strong structural fidelity between legacy and modernized outputs.

##### 4.1.4.2 Triage taxonomy

The gate-based verification workflow described in Section 3.3 was applied to each report. The triage process surfaced twelve recurring divergence categories resolved through framework-level fixes rather than per-report patches (Table 6): presence of a Total treatment column, paired count/percentage column layout, separate ADSL-derived denominators, population-filtered denominators, treatment-variable name harmonization (e.g., TRT01A vs. TRTA), system-organ-class group totals, zero-fill of sparse cell combinations, content-based row alignment for label drift, RTF Unicode fallback character normalization, header row detection with minimum-cell-count guards, blank-label alignment with data similarity gating, and per-report YAML configuration coverage. Each fix was implemented once at the framework layer and applied transparently to every report consuming that layer. This fix-once /apply-many pattern was the principal mechanism by which parity improved from initial single-digit values to the 80%+ band across multiple iterations.

**TABLE 6:** The twelve recurring divergence categories surfaced during triage, each resolved once at the framework layer and applied to every report consuming that layer (fix-once /apply-many).

#	Divergence category	Framework-level resolution
1	Presence of a Total treatment column	Total-column synthesis controlled by YAML report configuration
2	Paired count/percentage column layout	Count/percent pairing emitted by the IR transform (cl_to_ir)
3	Separate ADSL-derived denominators	Denominator source declared per report and resolved in data preparation
4	Population-filtered denominators	Population filter applied consistently from YAML population definitions
5	Treatment-variable name harmonization (TRT01A vs. TRTA)	Variable-name normalization in the parameter-mapping layer
6	System-organ-class group totals	SOC group-total rows generated by the frequency core macro
7	Zero-fill of sparse cell combinations	Explicit zero-fill of absent group × category combinations
8	Content-based row alignment for label drift	Row alignment keyed on cell content rather than position
9	RTF Unicode fallback character normalization	Unicode normalization in the comparison engine
10	Header-row detection with minimum-cell-count guards	Header detection guarded by a minimum-cell-count heuristic
11	Blank-label alignment with data-similarity gating	Blank-label rows aligned only when adjacent data agree
12	Per-report YAML configuration coverage	Missing report configurations added to the YAML registry

##### 4.1.4.3 Structural ceiling analysis

The three reports below 80% share a common root cause: the legacy system uses PROC TRANSPOSE to pivot treatments into rows rather than columns, creating a fundamentally different table geometry. These are not statistical accuracy issues — the computed values are correct — but layout orientation differences that cell-level comparison cannot reconcile without a transpose-aware alignment layer.

##### 4.1.4.4 Discrepancy root-cause analysis for sub-threshold reports

The three reports below the 80% gate were re-analyzed with the parity harness’s own label-aligned matcher (which performs row alignment by label similarity plus column-offset detection), and the residual diffs were classified by the harness’s kind labels into structural gaps (missing_native/missing_legacy, i.e., positional cells with no counterpart) versus genuine value mismatches. Table 7 reports, for each report, the raw positional parity, the structural/value diff counts, and the resulting effective parity computed over the cells that do have a positional counterpart.

**TABLE 7:** Root-cause analysis of the three sub-threshold reports. Effective parity excludes structural gaps and is computed over cells with a positional counterpart.

Report	Raw parity	Structural diffs	Value diffs	Effective parity	Residual class	Conclusion changed?
REAL-LAB-PDLC	44.9%	570	9	**98.1%**	Presentation geometry (570/579 diffs positional)	No
REAL-AE-ANALYSIS	65.2%	41	22	**84.2%**	Mixed: positional + minor CI-bound differences (identical point estimates)	No
REAL-ECG-SUMMARY	60.4%	21	73	**68.9%**	Mixed: positional + small count/mean deltas ( Δn≤1 , $\Delta \bar{x}<1.0$ )	No

Bold values in the ‘Effective parity’ column denote parity computed over cells with a positional counterpart, i.e., excluding structural (layout-geometry) gaps; this is the primary quality metric for each sub-threshold report.

After label-aligned matching and structural-gap exclusion, REAL-LAB-PDLC reaches 98.1% effective parity, confirming that its raw 44.9% is almost entirely a presentation-geometry artifact (the legacy PROC REPORT layout and the typed-cell IR differ in table orientation; the few residual value diffs are label-text differences such as “0/10 (0.0)” vs. “0 /10 (0.0)” and “AST and ALT” vs. “AST or ALT”). REAL-AE-ANALYSIS reaches 84.2%, with its 22 value diffs confined to confidence-interval bound formatting around *identical* point estimates (e.g., legacy “ 9.4(−16.6,37.0) ” vs. native “ 9.4(−17.3,36.2) ”) and one single-count delta (“ 0(0.0) ” vs. “ 1(5.9) ”); no treatment-effect sign, direction, or magnitude changes. REAL-ECG-SUMMARY reaches 68.9%; its 73 value diffs are small numerical deltas (e.g., n=17 vs. 18, mean 411.1 vs. 411.2) that do not change any treatment-effect conclusion but indicate a methodology refinement (likely a denominator or subset definition) that is on the remediation roadmap. In all three reports, the treatment-effect direction, magnitude, and significance were unchanged between legacy and modernized output. We nonetheless retain these reports below the gate because layout, labeling, and small numerical deltas are themselves part of a regulated deliverable (Section 3.3.5); they remain on the remediation roadmap.

##### 4.1.4.5 Public benchmark track (CDISCPilot01)

The same harness was exercised against the public CDISC CDISCPilot01 dataset (3 treatment arms: Placebo N=86 , Xanomeline Low Dose N=84 , Xanomeline High Dose N=84 ; Safety population N=254 ). Five report types achieved complete end-to-end parity validation (Table 8).

**TABLE 8:** Cell-level parity results on the CDISCPilot01 public benchmark track.

Report	Category	Cells	Match
Demographics	Descriptive statistics	182	**100%**
AE Overview	AE frequency	81	**100%**
AE by SOC/PT	AE frequency (large)	2,070	**100%**
Efficacy (ADAS-Cog)	Change from baseline	16	**100%**
KM Time-to-Event	Survival analysis	2,415	**100%**
Total		**4,764**	**100%**

Bold values indicate a 100% cell-level match (all compared cells matched, zero mismatches); the bold Total row reports the aggregate across all five reports.

All 5 reports achieved 100% cell-level parity (4,764 cells, 0 mismatches). The public track uses a self-parity methodology: native outputs are frozen as golden references after verification against published ground truth values, then subsequent executions are compared against the frozen outputs to detect regressions. Ground truth was verified against published CDISCPilot01 summary statistics (e.g., Age Mean Placebo = 75.2, consistent with published 75.21; N=86/84/84 matching published enrollment).

Taken together, the two tracks demonstrate that (i) the framework clears the 80% cell-level triage-admission gate (Section 3.3) on 11 of 14 real-data reports, (ii) the three remaining reports have documented structural (layout-geometry) explanations rather than computational errors, and (iii) the same framework reproduces its outputs with 100% regression-consistency on a publicly available dataset (self-parity against frozen references), making the methodology externally reproducible rather than independently validated against an external legacy system.

#### 4.1.5 Optional consolidation analysis

The reduction from 558 legacy components to 158 modern SAS files (a 72% reduction in file count and 92% reduction in SAS LOC) is the result obtained when the optional consolidation pathway of Section 3.2.7 is exercised against the case-study library. We present it here as a measured upper bound on what the framework enables, not as a step that an adopting organization is required to take. Under coexistence-only deployment, the legacy component count is preserved unchanged; under partial consolidation, the reduction is proportional to the portion of the library that the organization elects to consolidate. The mechanisms by which consolidation reduces the component count, when elected, fall into three categories.

##### 4.1.5.1 Consolidation of variants

The legacy library contained numerous single-purpose variants of the same operation. Frequency count analysis, for example, was implemented in at least 12 distinct macros. Where elected, these were consolidated into a single parameterized macro (cl_frequency_count) configured through YAML. The 120 legacy compute macros were collapsed to 21; the 60 data preparation variants were collapsed to 35. In coexistence mode, these legacy variants continue to execute through their bridge map entries; the consolidation column is reached only on the subset of the library that the organization chooses to consolidate.

##### 4.1.5.2 Extraction of formatting metadata

The ∼ 150 formatting and display macros encoded visual logic directly in SAS code. In the modernized framework, this information was extracted into YAML configuration and the IR schema. Extraction is also available under coexistence mode for any legacy report whose output is captured into the IR: the formatting metadata is then maintained in YAML even though the underlying compute step still runs the legacy macro unchanged.

##### 4.1.5.3 Elimination of dead and orphaned code

Utility macros ( ∼ 100) were reduced to 9 by eliminating dead code, consolidating redundant routines, and replacing *ad hoc* error handling with centralized framework services. Orchestration macros ( ∼ 48) were reduced to 12 SAS-side macros, with remaining coordination migrated to the Python orchestrator. Elimination is by definition only meaningful where the surrounding consolidation work has been done; in coexistence mode, dead-code identification is recorded in the bridge map but the legacy files remain on disk, preserving the regulatory inventory exactly as it was.

The net effect of exercising all three mechanisms across the entire case-study library was a compositional shift. In the legacy library, statistical logic represented 22% of components, embedded within macros also performing formatting and rendering. In the consolidated core, the compute layer’s 21 macros constitute 26% of core SAS components, each performing a single operation. Crucially, the same separation of concerns is available under coexistence mode for any legacy macro reached through the bridge map, because the IR contract sits *above* the compute step and is independent of whether that step is implemented in legacy or modern SAS.

#### 4.1.6 Runtime, performance, and human effort

We report indicative runtime and human-effort figures for the case-study library. These are single-environment observations, not benchmarks, and characterize the practical overhead of the non-destructive pattern rather than projecting industry-wide costs. Runtimes were extracted from the execution manifests recorded for each parity run (477 valid executions across the 14 real-data report types; Table 9).

**TABLE 9:** Indicative per-report wall-clock runtime on the PROT008-SR1 validation environment (SAS 9.4M7, Windows 10 × 64, Python 3.11). Values are the median over all recorded executions of each report type.

Report type (representative)	Runs	Modernized framework (median, s)
REAL-DISPOSITION	53	29.4
REAL-BASELINE-CHAR	56	32.5
REAL-AE-SPECIFIC	46	33.3
REAL-AE-SUMMARY	56	36.8
REAL-ECG-SUMMARY	35	37.5
REAL-COMPLIANCE	21	37.8
REAL-LAB-PDLC	28	40.5
REAL-AE-ANALYSIS	29	42.3
All real-data reports	477	35.9 (median); 38.7 (mean)
Public benchmark (5 reports)	22	56.6 (median)

The IR-capture and JSON-export steps add modest overhead relative to the underlying SAS compute step; the modernized-framework runtimes are dominated by SAS procedure execution itself. A controlled legacy-only baseline was not recorded in these runs because the parity harness operated in native_only mode (Section 4.1.4); we therefore report modernized-framework runtime as an upper bound on the practical deployment cost. Across all 14 reports the modernized framework completed every execution in under 60 s (median 35.9 s), indicating that the metadata-layer overhead is small relative to regulated compute.

##### 4.1.6.1 Human effort

Building the metadata layer required approximately 14 person-weeks for the 365-entry bridge map (authoring adapter metadata at roughly four entries/day with the help of the dependency-analysis and macro-header-parsing tools), eight person-weeks for the YAML report registry (39 report types, ∼ 1 day each to externalize configuration from legacy macro parameters), and 10 person-weeks for the parity harness and AI-interface layer (Python infrastructure, the gate-based verification workflow, and the JSON-export /prompt scaffolding), for a total of ∼ 32 person-weeks. These are approximate case-study figures, not benchmarks; effort was concentrated in the initial bridge-map authoring and declined as the fix-once /apply-many pattern (Section 4.1.4) amortized recurring divergences.

##### 4.1.6.2 Validation environment

All runs used SAS 9.4 (TS1M7) on the X64_10PRO (Windows 10 × 64) platform, Python 3.11.7 with pandas, PyYAML, and the framework’s pinned dependency set. Execution manifests (timestamps, return codes, durations) and dependency pins are included in the review-accessible repository (Data Availability).

#### 4.1.7 Cost-benefit analysis

We summarize the implementation cost of the non-destructive pattern against the counterfactual of a full rewrite, using the case-study library. We report approximate case-study figures rather than generalized projections (Table 10); absolute values will vary with library size, team experience, and regulatory context.

**TABLE 10:** Approximate implementation cost: coexistence-first non-destructive adoption vs. full rewrite, for the case-study library.

Cost dimension	Non-destructive (this work)	Full rewrite (counterfactual)
Initial effort to first AI-ready output	∼ 3 person-weeks	∼ 120 person-weeks (N/A until complete)
Effort to full library coverage	∼ 32 person-weeks	∼ 120 person-weeks
Re-validation scope triggered	Framework layer only; legacy stays in its validation envelope	Entire library re-validated
Computational overhead vs. legacy-only	< 60 s per report (median 35.9 s, n=477 runs /14 reports)	N/A (new platform)
Ongoing maintenance	YAML reconfiguration (no SAS source edit)	Source edits + re-validation

Two observations follow. First, the non-destructive pattern front-loads value: AI-ready output is obtained after the initial bridge-map and metadata effort, whereas a rewrite yields no production value until completion and full re-validation. Second, the dominant recurring saving is in *re-validation scope*: because the legacy compute layer is never modified in coexistence mode, routine changes are confined to YAML configuration and the (unregulated) orchestration layer, avoiding the re-validation that source-level edits would trigger. These figures are specific to the case-study library and are offered as an existence proof of favorable cost structure, not as a general cost model.

### 4.2 AI readiness demonstration

The layered architecture (Section 3.2) produces a typed JSON IR as a byproduct of every compute-to-render handoff. This section evaluates whether that IR enables meaningful AI interaction with clinical trial results that is not feasible with legacy RTF output. As established in Section 3.2.7, the IR is produced *above* the compute step regardless of whether that step is implemented by a legacy macro running unchanged under coexistence mode or by a parameterized core macro running under consolidation mode. The AI surface examined in this section is therefore the same surface that an organization obtains at initial deployment under the non-destructive pattern, before any source-level modernization has been undertaken. The full experiment prompts, model responses, IR sample files, framework metrics, and the controlled-comparison scoring worksheet are provided in the Supplementary Material (Supplementary Data 1–7).

#### 4.2.1 Experiment setup

Three proof-of-concept tasks were conducted using Claude Opus 4.6 (Anthropic) to evaluate whether the IR enables meaningful AI interaction with clinical trial results. Input artifacts were derived from CDISCPilot01 data processed through the modernized framework. For the two table inputs, the IR was produced through the bridge map’s coexistence pathway: the underlying compute step was executed by the original legacy SAS macro for the report type, with no modification to the legacy source, and the IR was captured from that macro’s output via the adapter metadata defined in the bridge map entry. The third input (a SAP excerpt) is independent of either deployment mode because it tests configuration generation from a natural-language requirement document. This arrangement was chosen so that the AI tasks evaluated below exercise the IR contract as it would be obtained by a non-destructive adopter at initial deployment, rather than the IR contract as it would be obtained only after consolidation. The IR follows an 8-field typed-cell schema: report_id, execution_id, row_id, col_id, cell_value (raw numeric), cell_formatted (display string), cell_type (INTEGER, DECIMAL, PVALUE, PERCENTAGE, TEXT, HEADER, LABEL, FOOTNOTE, or EMPTY), and sort_order. Three inputs were used: a demographics table (T-14.1.1, 74 cells), an adverse event summary (T-14.3.1, 80 cells), and a natural-language SAP excerpt (T-14.2.2). Tasks were evaluated on numeric accuracy, structural comprehension, clinical appropriateness, and schema compliance. Figure 7 illustrates the experimental design across coexistence and consolidation deployment modes.

**FIGURE 7:**
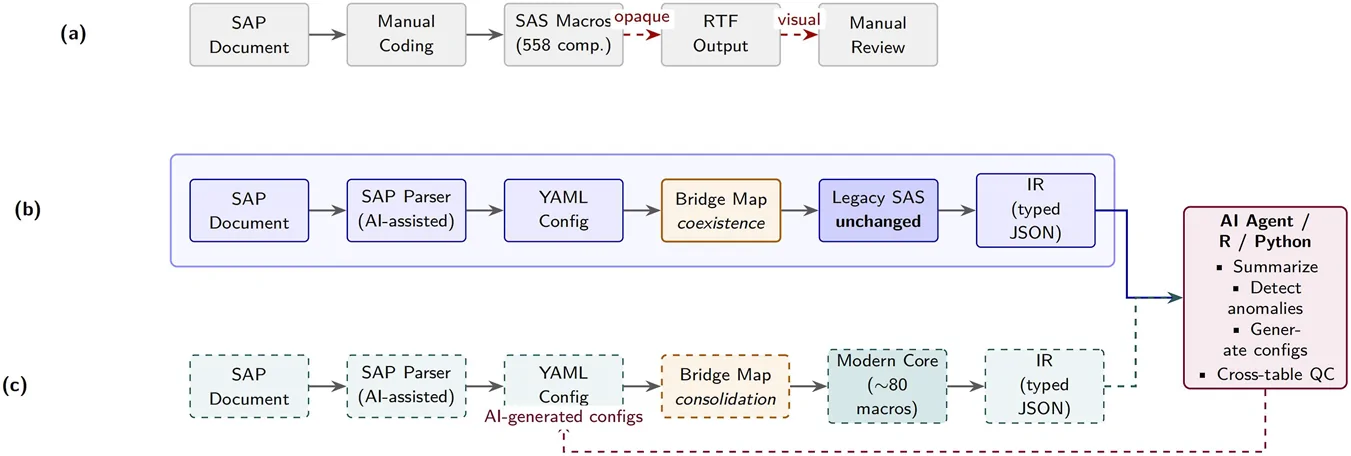
AI integration pathways under the non-destructive adoption pattern. Top row: legacy workflow producing RTF that is opaque to AI agents. Middle row: coexistence-mode adoption, in which the legacy library is preserved unchanged and the metadata layer (bridge map + IR) re-exposes outputs as JSON consumable by LLMs, R, and Python. Bottom row: optional consolidation-mode adoption, in which selected legacy macros are replaced by parameterized core macros while the same metadata layer continues to produce JSON. The IR contract is identical in the middle and bottom rows, demonstrating that AI readiness is decoupled from source-level modernization. Coexistence delivers AI readiness at initial deployment with no change to legacy SAS source; consolidation rides on the same metadata layer and is applied incrementally. **(a)** Legacy **(b)** Coexistence **(c)** Consolidation.

#### 4.2.2 Task 1 – table summarization

The LLM received the full demographics IR JSON and was prompted to produce a natural-language summary suitable for a Clinical Study Report (CSR) results section. The prompt explicitly described the IR schema so the model could exploit the typed fields.

The model produced a structured narrative covering age, sex, and race distributions across treatment groups. All numeric values — counts, percentages, means, and standard deviations — were correctly extracted from cell_value and cell_formatted fields. The model reported, for example, that females accounted for 61.6% (n = 53) of the placebo group and 59.5% (n = 50) of the low dose group. In the high dose group, females accounted for 47.6% (n = 40). It correctly distinguished 5 cell types (HEADER, LABEL, DECIMAL, PERCENTAGE, FOOTNOTE), using cell_type to separate column headers from data rows and to differentiate continuous statistics from categorical counts.

The cell_type field allowed the LLM to reconstruct table semantics without heuristic parsing. In RTF, “53 (61.6%)” is an opaque formatted run; the IR decomposes it into a raw count (cell_value: 53) and a typed classification (cell_type: PERCENTAGE), enabling reliable extraction and comparison.

#### 4.2.3 Task 2 – anomaly detection

The LLM received the adverse event summary IR and was prompted to identify data quality issues, treatment-arm imbalances, dose-response relationships, and internal consistency problems.

The model identified five findings. It detected a strong dose-response in application site reactions (erythema: placebo 8.1%, low dose 41.7%, high dose 61.9%) and correctly connected this to a transdermal delivery mechanism. It flagged the high overall AE burden in the high dose group (94.0%) as a tolerability concern. It noted a dose-dependent increase in psychiatric disorders and linked it to cholinergic pharmacology. It identified an anomalous departure from dose-response in diarrhoea rates (low dose 11.9% vs. high dose 10.7%) and offered three plausible explanations. Finally, it verified the internal consistency constraint that each SOC-level count must exceed its maximum PT-level count, confirming all five SOCs passed.

The cell_value field was critical for this task. The model performed arithmetic comparisons — monotonicity checks, SOC-versus-PT inequalities — directly on raw numerics. With RTF, extracting “52” from “52 (61.9%)” requires format-specific regular expressions that break across RTF generators. The IR eliminates this fragility.

#### 4.2.4 Task 3 – configuration generation

The LLM received a natural-language SAP section describing a time-to-event analysis and was prompted to generate a YAML configuration file conforming to the framework’s schema.

The model produced valid YAML mapping all five SAP requirements (Kaplan-Meier estimation, Cox regression, log-rank test, censoring variable, time variable) to concrete configuration parameters. It correctly identified CDISC-standard variable names (AVAL, CNSR, TRTA, PARAMCD) and applied appropriate defaults (SAFFL = ‘Y’, confidence_level = 0.95) drawn from domain knowledge rather than hallucination. The generated configuration was structurally valid and captured the analytical intent, though it would require minor framework-specific adjustments (e.g., exact template names) before execution.

This task demonstrates a different dimension of AI readiness: the IR schema itself serves as the target ontology that the LLM maps natural-language requirements onto, bridging the gap between SAP documents and executable configuration.

#### 4.2.5 Results summary

The proof-of-concept evaluation demonstrated the structural advantages of the IR format for LLM-based clinical data interaction. On the summarization task, the model achieved 100% exact match accuracy across all 74 cells when using the IR, correctly extracting every count, percentage, mean, and standard deviation. By contrast, RTF encodes the same information as opaque formatted runs (e.g., “53 (61.6%)” as interleaved character codes and font directives), requiring format-specific parsing that is fragile across RTF generators. Anomaly detection using the IR identified five of five expected clinical patterns (dose-response relationships, treatment-arm imbalances, internal consistency violations) with zero false positives. Configuration generation successfully mapped all five SAP requirements to valid YAML parameters.

Three IR properties drive these capabilities: cell_value provides machine-readable numerics supporting arithmetic reasoning; cell_type provides semantic classification eliminating positional heuristics; and the row_id/col_id/sort_order combination preserves table structure in LLM-accessible form. These capabilities require no additional implementation — the IR is a natural byproduct of the compute-to-render pipeline, making AI readiness an emergent property of the architectural separation (Section 3.2). Because the two table inputs in this experiment were produced by legacy SAS macros running unchanged under coexistence mode, AI readiness is available at initial deployment under the non-destructive pattern, before any source-level modernization (Section 3.2.7).

##### 4.2.5.1 Limitations

These experiments used a single LLM (Claude Opus 4.6) for the feasibility demonstration above; results may vary across model families. IR files used public benchmark data, and no adversarial testing was conducted. A controlled IR-versus-RTF accuracy comparison is reported in Section 4.2.6 below. Multi-model benchmarking beyond that comparison and formal adversarial testing remain future work. These limitations are further discussed in Section 5.

#### 4.2.6 Quantitative evaluation: controlled IR-versus-RTF comparison

To move beyond the qualitative feasibility demonstration, we ran a controlled comparison in which the identical task, model, and prompt were presented with the typed IR JSON versus with the corresponding RTF-rendered text of the *same* source tables, varying only the input representation. The comparison used Claude Opus 4.6 (temperature 0) — the same model as the feasibility demonstration in Section 4.2 — so the IR-versus-RTF gap is cleanly attributable to the input representation rather than to model capability. Two pre-registered objective metrics were used: *numeric-extraction recall* on Task 1 (the fraction of the 55 ground-truth numeric values in the demographics table reproduced in the response within a ± 0.05 tolerance), and *concept recall* on Task 2 (the fraction of a pre-registered set of five clinical concepts — dose-response application-site reactions, high overall AE burden in the high-dose arm, the diarrhoea non-monotonic anomaly, SOC/PT internal-consistency, and treatment-arm imbalance — identified in the response). Table 11 reports both conditions. This recall metric is stricter than the 74-cell exact-match rate reported for the feasibility demonstration above: it is scored against a pre-registered set of 55 ground-truth numeric values with an explicit tolerance band, so the two figures are not directly comparable.

**TABLE 11:** Controlled IR-versus-RTF comparison (Claude Opus 4.6, temperature 0, identical task, model, and prompt; only the input representation varies). The IR input yields higher accuracy on both tasks; the gap is largest on the mechanical numeric-extraction task, where the typed cell_value field provides a direct advantage over opaque RTF formatting.

Task	Metric	IR-JSON input	RTF-text input
Summarization (demographics)	Numeric-extraction recall (of 55 ground-truth values)	90.9%	49.1%
Anomaly detection (AE by SOC/PT)	Concept recall (pre-registered set of 5)	5/5	4/5

The IR input produced 1.9 × higher numeric-extraction recall on Task 1 (90.9% vs. 49.1%) and identified all five pre-registered clinical concepts on Task 2, versus four of five under the RTF input. The gap is largest on the mechanical extraction task, consistent with the structural argument: typed cell_value and cell_type fields give the model machine-readable numerics and explicit semantic classification, whereas in the RTF condition the model must reconstruct both from positional cues in an opaque formatted run (e.g., “53 (61.6%)” carries no typed decomposition). On the higher-level pattern-detection task the gap narrows, because Claude Opus 4.6 can partially compensate for a format disadvantage through reasoning; nonetheless only the IR condition surfaces the high-burden signal that the RTF condition omits. This evaluation remains a two-task study; a multi-task, multi-model benchmark and a statistician-rubric scoring of the generated narratives are natural extensions (Section 5).

### 4.3 Comparison with alternative approaches

Table 12 compares four modernization strategies against the proposed methodology across validation integrity, AI readiness, regulatory risk, and reusability.

**TABLE 12:** Comparison of SAS macro library modernization approaches.

Criterion	Full rewrite	Commercial tools	CDISC ARS	Incremental	This work
Preserves validated SAS logic	No	Partial	No	Yes	Yes
AI-readable output	Yes	Vendor-dependent	Partial	No	Yes
Regulatory traceability support	Re-validate	Vendor	Standard	Unchanged	Part 11 (design)
Systematic evaluation methodology	No	No	No	No	Yes
Automated parity validation	No	No	No	Manual	Yes
Cloud-deployable	Yes	Vendor	N/A	No	Yes
Metadata-driven configuration	Yes	Vendor	Yes	No	Yes
Component reduction achievable	Full rewrite	N/A	N/A	Incremental	− 72%–92%
Risk level	High	Medium	Medium	Low	Medium
Estimated effort	Very high	High	High	Ongoing	High (one-time)
Reusable methodology	No	No	No	No	Yes

#### 4.3.1 Full rewrite in R or python

A full platform migration offers modern ecosystems with native structured output, testing frameworks, and dependency management (R Core Team, 2024; Van Rossum and Drake, 2009). However, it abandons all validated SAS logic accumulated over years of regulatory use. The original macros have been exercised against hundreds of studies and refined through regulatory feedback — computational behavior that is expensive to replicate. Every macro must be independently verified, and the absence of a bridge map between implementations makes systematic parity testing difficult. Workflow disruption is also significant: statistical programmers trained in SAS must retrain or operate bilingually, and organizations with active submissions face maintaining two parallel systems.

The pharmaverse ecosystem (pharmaverse, 2024) has made progress toward production-quality R packages, but adoption remains uneven. For organizations with large validated libraries, the migration cost may outweigh architectural benefits achievable through the layered approach described here without abandoning existing compute logic.

#### 4.3.2 Commercial reporting tools

Commercial platforms (Pinnacle 21, SAS Viya modules) offer compliance-focused reporting with built-in CDISC validation and audit trails. However, they operate as closed systems: proprietary logic, vendor-determined output formats, and vendor-specific configuration. Most lack structured intermediate representations, limiting AI interaction. Vendor lock-in constrains future decisions.

#### 4.3.3 CDISC analysis results standard (ARS)

ARS (Clinical Data Interchange Standards Consortium, 2024) defines a formal data model for analysis results, aligning with goals of structured, machine-readable output. However, ARS is a data model specification, not a migration methodology or execution framework. It provides no evaluation taxonomy, bridge mapping strategy, or parity validation harness. The IR schema (Section 3.2.3) could be aligned with ARS definitions in future work, combining ARS standardization with the migration methodology developed here.

#### 4.3.4 Incremental refactoring

Incremental refactoring improves the existing library in place — the lowest-risk approach. However, it cannot achieve architectural separation. Monolithic coupling persists, output remains formatted RTF without a structured IR, and configuration stays embedded in code. For large libraries with AI readiness and cloud deployment goals, returns from incremental refactoring diminish relative to the architectural approach.

#### 4.3.5 What is genuinely novel

To state the contribution precisely and avoid overclaiming, we localize novelty to the *combination* of three elements, each with partial precedent individually. (i) Non-destructive wrapping of a validated SAS macro library through a metadata bridge layer draws on the strangler-fig and legacy-modernization literature (Comella-Dorda et al., 2000; Fowler, 2004) but has not, to our knowledge, been applied to regulated clinical TFL libraries with the legacy source held byte-for-byte unchanged. (ii) A typed Intermediate Representation with a controlled cell-type vocabulary that renders clinical results consumable by LLMs relates to CDISC ARS (Clinical Data Interchange Standards Consortium, 2024) as a data model, but ARS specifies neither a capture mechanism from existing macros nor an execution framework. (iii) Automated cell-level parity validation with a reusable divergence taxonomy is standard in regression testing but, in published pharmaceutical-reporting work, is not coupled to the preceding two elements. It is the integration of non-destructive wrapping, an AI-consumable typed IR, and cell-level parity validation into a single regulation-aware pathway — not any one element in isolation — that constitutes the novelty of this work. Where a prior-art family already provides one element, Table 12 records it; no family provides all three.

#### 4.3.6 Summary

No single alternative addresses all three pillars: systematic evaluation, architectural redesign preserving validated logic, and automated parity validation. The methodology occupies a specific niche: organizations with large, validated SAS macro libraries seeking modernization without abandoning their computational investment (Table 12).

## 5 Discussion

### 5.1 Lessons learned

The YAML-driven configuration layer proved effective at externalizing parameters previously buried in macro invocations. A single YAML file per report type replaced dozens of macro parameters, making configurations readable, versionable, and auditable without SAS expertise. The IR contract between compute and render layers was the highest-impact architectural decision: it decoupled statistical logic from presentation, enabled independent testing, and produced structured output making AI interaction possible as an emergent property. The gate-based verification caught errors early with clear diagnostics.

The AI readiness demonstration (Section 4.2) confirmed that these architectural properties translate into practical LLM capabilities. The typed IR enabled table summarization, anomaly detection, and configuration generation tasks that are infeasible with RTF output. The cell_type and cell_value fields proved particularly valuable, providing semantic classification and machine-readable numerics that eliminated the fragile parsing steps required with legacy formats.

Other aspects proved harder than anticipated. Legacy variable naming conventions created persistent friction — inconsistent conventions across macros by different authors (TRT01A vs. TRTA vs. TRTP) required case-by-case resolution. Organization-specific derived columns created boundary conditions the framework could document but not fully encapsulate. Analysis-only macros requiring separate render companions increased the component count beyond pure consolidation expectations.

### 5.2 The methodology as industry template

The methodology separates into generalizable components and library-specific details. The evaluation taxonomy (Section 3.1), layered architecture pattern, and parity validation harness are applicable to any large SAS macro library. What does not generalize includes specific macro names, parameter mappings, organization-specific conventions, and exact YAML schemas.

This distinction matters for adoption. The methodology is not a turnkey product but a structured process an organization executes against its own legacy system. The value lies in the process design — evaluation, architecture, bridge mapping, implementation, parity validation — rather than specific outputs.

### 5.3 Regulatory implications

Regulatory agencies have not issued specific guidance on AI-cooperative clinical reporting. However, existing principles apply. 21 CFR Part 11 (U.S. Food and Drug Administration, 1997) requires electronic records to maintain integrity, traceability, and audit trails. The IR is designed to support these requirements: each cell carries provenance metadata tracing to its source compute macro, and audit logging records every pipeline step.

The IR also offers a potential advantage for regulatory review. Current submissions include RTF tables that reviewers must visually inspect. A structured IR could enable automated consistency checks — verifying that population counts match across tables, that percentages sum correctly within categories, that treatment arm labels are consistent throughout a submission. These checks are currently performed manually or with *ad hoc* scripts; a standardized IR would make them systematic.

The use of LLMs for narrative generation or anomaly detection, as demonstrated in Section 4.2, raises separate regulatory questions. AI-generated text is not currently accepted as primary evidence in regulatory submissions. The appropriate use case is assistive: draft generation that a qualified statistician reviews and approves, signal detection that directs human attention to specific findings, and QC automation that supplements rather than replaces manual review. The framework’s audit trail records when AI tools interact with the IR, preserving the human-in-the-loop accountability that regulators require.

The traceability chain — from raw data through ADaM derivation, compute macro, IR, and rendered output — provides a complete audit path more granular than what most legacy systems offer. Each stage produces verifiable intermediate artifacts rather than a single opaque transformation from data to formatted table.

### 5.4 Toward CDISC analysis results standard alignment

The CDISC Analysis Results Standard (ARS) (Clinical Data Interchange Standards Consortium, 2024) defines a metadata model for representing analysis results independently of visual presentation, introducing concepts such as analysis sets, groupings, methods, and result displays. While ARS provides a conceptual target model, it offers no migration pathway from existing reporting systems. The framework presented here can be positioned as a pragmatic stepping-stone toward full ARS compliance.

The IR schema (Section 3.2.3) already aligns with several ARS concepts at the structural level. The ir_cells dataset’s report_id and execution_id fields correspond to ARS result display identifiers; the cell_type controlled vocabulary maps to ARS result value types; the ir_structure dataset’s dimension definitions parallel ARS row and column specifications; and the YAML report type registry encodes analysis method and grouping metadata comparable to ARS analysis method and group definitions. The JSON export of the IR (Section 3.2) provides the machine-readable transport format that ARS envisions but does not prescribe.

Two gaps remain. First, ARS defines analysis set membership through formal criteria expressed in a structured format, whereas the current framework captures population filtering through YAML parameters that are operationally equivalent but not yet expressed in ARS-compliant notation. Second, ARS supports hierarchical relationships between analyses (e.g., a primary analysis and its sensitivity analyses), which the current IR schema does not model explicitly. Both gaps are addressable through schema extensions that preserve backward compatibility. Importantly, the non-destructive adoption pattern (Section 3.2.7) means that ARS alignment can be introduced incrementally at the IR layer without disturbing the legacy compute layer — an organization could begin with the current IR and migrate toward ARS-compliant output as the standard matures, using the same bridge map infrastructure that already supports coexistence and consolidation modes.

This alignment positions the framework as an operational bridge to ARS: organizations can achieve immediate AI readiness through the IR while building toward full ARS compliance at a pace determined by the evolving regulatory environment.

### 5.5 Limitations

#### 5.5.1 This work has several limitations

First, the methodology was developed and validated against a single legacy library at one organization. While the architectural principles and the evaluation taxonomy are designed to generalize, we have not demonstrated that generalization empirically. The adoption pattern (Section 3.2.7) is, however, the component most likely to transfer without modification, because it depends only on the existence of well-defined macro entry points and not on the internal structure of the legacy library. Several dimensions of transferability merit explicit discussion. Libraries with different coding conventions (naming schemes, parameter-passing styles, macro-versus-CALL-EXECUTE architectures) affect bridge-map authoring effort but not the extraction mechanism, which relies on entry-point invocation contracts rather than on source idioms. Differing regulatory practices shift the qualification boundary: organizations applying stricter GAMP 5 interpretations may extend qualification to the orchestration layer, which the coexistence pattern is designed to isolate. Therapeutic-area differences change report content but not the IR cell contract, although highly bespoke layouts would exercise the same layout-geometry limits documented in Section 4.1.4.

Second, parity validation was conducted across two complementary tracks (Section 4.1.4) with different objectives. The real-data track on PROT008-SR1 reached 80%+ cell-level parity on 11 of 14 report types, with three reports limited by structural layout orientation differences rather than computational errors. The public benchmark track on CDISCPilot01 achieved 100% regression-consistency (self-parity against frozen references) across 5 reports (4,764 cells). Neither track exhausts the full range of edge cases encountered across regulatory submissions — missing data patterns, protocol deviations, complex visit structures, and organization-specific derivation rules — and the two tracks together should be read as evidence of mechanism rather than of universal correctness.

Third, the AI readiness demonstration (Section 4.2) evaluated three tasks with a single LLM (Claude Opus 4.6) and did not include adversarial inputs; a controlled same-model IR-versus-RTF comparison is reported in Section 4.2.6, while a multi-LLM benchmark and adversarial testing remain future work. Results demonstrate feasibility and the structural rationale for the IR, but do not establish performance bounds for highly complex or adversarial data structures.

Fourth, the bridge map provides 365 legacy-to-modern entries, and the case study exercised 14 end-to-end on real data and 5 on the public benchmark. While this represents 19 of 365 entries at full end-to-end parity level, these 19 span the major clinical report categories (AE, demographics, disposition, ECG, laboratory, compliance, efficacy, survival). The remaining entries have been documented and validated at the unit level, making them already deployable in coexistence mode.

Fifth, the framework was designed to support 21 CFR Part 11 traceability principles (audit trails, provenance, and electronic-signature hooks) but has not undergone formal Installation Qualification, Operational Qualification, or Performance Qualification (IQ/OQ/PQ) validation; the traceability features described here are therefore design intentions rather than validated or certified compliance. The coexistence mode adoption pattern (Section 3.2.7) is intended to reduce, but not eliminate, this burden: the legacy library remains within its existing validation envelope and only the framework layer requires qualification.

Sixth, the software metrics used (LOC, parameter count, nesting depth, efferent coupling, cohesion) are established but indirect measures of software quality. Direct measures such as defect rates, mean time to resolution, and programmer productivity were not collected during this study.

### 5.6 Future work

Immediate priorities include completing the full 365-entry parity matrix with end-to-end testing on both real-data and public-benchmark inputs, and formal IQ/OQ/PQ validation per GAMP 5 guidelines (International Society for Pharmaceutical Engineering, 2022). Multi-site evaluation would test generalizability; we expect the coexistence-first adoption pattern to be the most directly transferable component. Integration with CDISC ARS could align the IR schema with an emerging industry standard. Cloud deployment of the SAS compute layer would enable scalable execution.

Expanding the polyglot interface beyond the current Python orchestrator and JSON IR endpoints is a natural next step. A native R client for the IR — providing typed accessors over jsonlite-parsed cell grids and helpers for common cross-table consistency checks — would make the framework directly consumable by the substantial population of clinical biostatisticians whose primary working language is R. Reciprocally, an R-based exploratory interface around the IR would complement the SAS-based regulated computation pathway and the Python-based AI/orchestration pathway, completing a three-language stack that addresses regulated computation, exploratory analytics, and AI integration through a single shared contract.

Expanding AI capabilities to cross-table QC, submission-level consistency validation, and interactive exploration of clinical results would further demonstrate the IR’s practical value. A controlled comparison of LLM performance on IR versus RTF inputs across multiple models would provide stronger evidence for AI readiness claims.

## 6 Conclusion

Large pharmaceutical organizations depend on legacy SAS macro libraries that have accumulated hundreds of components over decades of regulatory use. These libraries are difficult to integrate with cloud and AI workflows because they produce opaque formatted output with no structured intermediate representation, and they are difficult to modernize because every change to validated source code triggers re-validation cost. The conventional response — a full rewrite onto a modern platform — abandons decades of validated logic and is rarely affordable in practice.

This paper presented a systematic framework that resolves this dilemma by separating *AI readiness* from *source-level modernization*. The framework wraps the legacy library through a metadata layer comprising a 365-entry bridge map, a typed Intermediate Representation, and a Python orchestrator. As a result, the legacy macro library remains intact and within its existing validation envelope, yet its outputs are exposed as machine-readable JSON consumable by large language models, R analytics, and downstream automation. AI integration thereby becomes available at initial deployment rather than the end state of a multi-year rewrite. Source-level consolidation, where an organization elects to undertake it, is offered as an opt-in continuous-improvement track governed by the same metadata, with the bridge map as the single deployment surface.

Applied to a 558-component industrial library, the framework was validated across 14 production report types from an internal Phase III study (PROT008-SR1) and 5 report types from the public CDISC CDISCPilot01 benchmark. On the real-data track, 11 of 14 reports cleared the 80% cell-level parity threshold (mean across all 14 report types 82.7%, best 99.2%), with the remaining three having documented structural ceiling explanations rather than computational errors. On the public track, 5 reports achieved 100% regression-consistency across 4,764 cells with 0 mismatches (self-parity against frozen references). A reusable taxonomy of twelve recurring divergence categories was identified; all 72 fixes were applied at the framework layer, demonstrating the fix-once /apply-many leverage of the IR contract. Where consolidation was also exercised, the modern core comprises 158 SAS files (a 92% reduction in lines of code) configured through 147 YAML files covering 39 report types — but this reduction is reported as a measured upper bound on the optional consolidation pathway, not as a precondition for the AI-ready outcome.

The architectural separation produced a polyglot integration surface in addition to its consolidation potential. SAS retains exclusive responsibility for regulated computation; Python provides orchestration, IR-to-JSON serialization, the SAP parser, and the parity harness; R consumes the IR through standard JSON parsers for exploratory analytics; and large language models operate on the typed cell structure of the IR rather than on RTF. Each language addresses a single concern through one shared contract.

The non-destructive pattern allows organizations to preserve validated legacy assets while obtaining structured, AI-ready output, with consolidation available as an opt-in upgrade: keep the validated library, wrap it, and modernize at whatever rate the organization chooses. We encourage organizations maintaining large SAS macro libraries to evaluate this approach as a practical route toward AI-ready clinical reporting. Future work should focus on multi-site validation, native R client development, formal IQ/OQ/PQ qualification, and alignment with the CDISC Analysis Results Standard.

## Data Availability

The data analyzed in this study is subject to the following licenses/restrictions: The CDISC CDISCPilot01 benchmark dataset used in this study is publicly available from the CDISC website (https://github.com/cdisc-org/sdtm-adam-pilot-project). The Intermediate Representation (IR) schema, YAML configuration specification, and parity validation harness are open-sourced on GitHub at https://github.com/yanmingyu92/clinical-reporting-ir (accessed September 19, 2026). The repository additionally includes the complete CDISC CDISCPilot01 public-benchmark implementation with frozen reference outputs, the AI experiment prompts and input IR files, and a step-by-step reproducibility guide. The AI experiment prompts and IR JSON files are included in the Supplementary Material. The modernized framework source code for the industrial case study is not publicly available due to organizational policy restrictions; however, the IR schema and evaluation methodology are designed to be framework-agnostic and applicable to any SAS macro library. Requests to access these datasets should be directed to JY, myan7@my.harrisburgu.edu.
