# Supplementary material for: A non-destructive methodological framework for modernizing legacy clinical reporting systems for AI-driven pharmacoinformatics: a SAS case study
Source: Front Pharmacol. 2026 Sep 28;17:1876207. doi: 10.3389/fphar.2026.1876207 (PMC13654759; doi:10.3389/fphar.2026.1876207)
Supplement: Supplementary file 1 [file DataSheet1.pdf]

# Supplementary Materials

## A Non-Destructive Methodological Framework for Modernizing Legacy Clinical Reporting Systems for AI-Driven Pharmacoinformatics: A SAS Case Study

Jaime Yan

## Contents

**1 Supplementary Data 1: AI Experiment Log** 2

**2 Supplementary Data 2: IR Demographics Sample JSON** 8

**3 Supplementary Data 3: IR Adverse Event Summary Sample JSON** 11

**4 Supplementary Data 4: SAP Time-to-Event Sample Text** 14

**5 Supplementary Data 5: Legacy Library Metrics** 15

**6 Supplementary Data 6: Modernized Framework Metrics** 16

# 1 Supplementary Data 1: AI Experiment Log

```
# AI Readiness Experiment Log

**Date:** 2026-04-24
**Framework:** Modernized Framework v5.0
**Model:** Claude Opus 4.6 (Anthropic)
**Objective:** Evaluate whether a typed JSON Intermediate Representation (IR) of clinical trial tables
    ↪ enables meaningful AI interaction that is impossible with legacy RTF output or raw SAS datasets.

---

## Input Artifacts

| File | Description |
|------|-------------|
| `ir_demographics_sample.json` | Demographics table IR (T-14.1.1), 74 cells, CDISCPilot01 Safety
    ↪ Population |
| `ir_ae_summary_sample.json` | AE summary table IR (T-14.3.1), 80 cells, top 5 SOCs |
| `sap_tte_sample.txt` | SAP excerpt for Table 14.2.2, time-to-event analysis |

---

## Experiment 1: Table Summarization

### Protocol

**Task:** Convert structured IR JSON into a natural-language summary suitable for inclusion in a Clinical
    ↪ Study Report (CSR) results section.

**Prompt sent to LLM:**
> Given the following JSON representation of a clinical trial demographics table, provide a natural-
    ↪ language summary suitable for a clinical study report. The JSON uses a typed-cell IR schema where
    ↪ each object has: report_id, row_id, col_id, cell_value (raw numeric), cell_formatted (display
    ↪ string), cell_type (INTEGER, DECIMAL, PERCENTAGE, LABEL, HEADER, FOOTNOTE, EMPTY), and sort_order.
    ↪  Reconstruct the table semantics from these fields and produce an accurate narrative.

**Input:** Full contents of `ir_demographics_sample.json` (74 cells).

### LLM Response

> **Summary of Demographics (Safety Population)**
>
> A total of 254 subjects were included in the safety population: 86 in the placebo group, 84 in the
    ↪ xanomeline low dose group, and 84 in the xanomeline high dose group.
>
> **Age.** The mean age was similar across treatment groups: 75.2 years (SD 8.59) in the placebo group,
    ↪ 75.7 years (SD 8.29) in the xanomeline low dose group, and 74.4 years (SD 7.89) in the xanomeline
    ↪ high dose group. The overall mean age was 75.1 years (SD 8.25). Age ranged from 51 to 89 years
    ↪ across all groups, with median values of 76.0 to 77.5 years.
>
> **Sex.** The study population was predominantly female. Females accounted for 61.6% (n=53) of the
    ↪ placebo group, 59.5% (n=50) of the low dose group, and 47.6% (n=40) of the high dose group. The
    ↪ high dose group had a notably higher proportion of males (52.4%) compared to placebo (38.4%) and
    ↪ low dose (40.5%).
>
> **Race.** The majority of subjects were White across all treatment groups (86.0%, 85.7%, and 88.1% for
    ↪ placebo, low dose, and high dose, respectively; 86.6% overall). Black or African American subjects
    ↪  comprised 9.1% of the total population (ranging from 7.1% to 10.7% across groups). American
    ↪ Indian or Alaska Native subjects comprised 4.3% of the overall population.
>
> Demographic characteristics were generally balanced across treatment groups, with a minor imbalance in
    ↪ sex distribution in the high dose group.

### Assessment

| Criterion | Rating | Notes |
|-----------|--------|-------|
| Numeric accuracy | PASS | All counts, percentages, means, and SDs correctly extracted from `cell_value`
    ↪ and `cell_formatted` fields |
| Structural comprehension | PASS | LLM correctly reconstructed the table hierarchy: header row identified
    ↪  treatment columns; LABEL cells identified parameter groups; PERCENTAGE cells parsed as "n (%)" |
```

```
| Clinical interpretation | PASS | Noted the sex imbalance in the high dose group without overstating its significance |
| cell_type usage | PASS | Distinguished HEADER (column identification), LABEL (row grouping), DECIMAL (continuous stats), PERCENTAGE (categorical stats), FOOTNOTE (metadata) |
| CSR-appropriate tone | PASS | Objective, quantitative, suitable for regulatory submission |

**Key observation:** The `cell_type` field allowed the LLM to distinguish section headers from data rows without heuristic parsing. The `cell_value` field provided machine-readable numerics that the LLM used for comparison (e.g., noting the high dose sex imbalance), while `cell_formatted` provided the display representation. This dual representation is impossible to extract reliably from RTF, where "53 (61.6%)" is an opaque string with no typed decomposition.

---

## Experiment 2: Anomaly Detection

### Protocol

**Task:** Identify data quality issues, unusual patterns, or clinically meaningful findings from AE summary IR data.

**Prompt sent to LLM:**
> Given the following JSON representation of an adverse event summary table from a clinical trial, identify any potential data quality issues, unusual patterns, or findings that warrant further investigation. The JSON uses a typed-cell IR schema. Focus on: (1) treatment-arm imbalances, (2) dose-response relationships, (3) unexpectedly high or low frequencies, and (4) any internal consistency issues.

**Input:** Full contents of `ir_ae_summary_sample.json` (80 cells).

### LLM Response

> **Anomaly Detection Report: AE Summary Table T-14.3.1**
>
> **1. Strong Dose-Response Pattern in Application Site Reactions**
>
> The most striking pattern is the clear dose-response relationship in application site events under General Disorders:
> - Application site erythema: Placebo 8.1% -> Low 41.7% -> High 61.9%
> - Application site pruritus: Placebo 5.8% -> Low 23.8% -> High 44.0%
> - Application site irritation: Placebo 3.5% -> Low 17.9% -> High 26.2%
>
> The magnitude of the increase from placebo to active treatment (5-8x for erythema) suggests these are drug-related local reactions consistent with a transdermal delivery system. The monotonic dose-response strengthens this interpretation.
>
> **2. Overall AE Burden Imbalance**
>
> The "Any Adverse Event" row shows a steep dose-response: Placebo 66.3% -> Low 85.7% -> High 94.0%. The high dose group has an AE rate approaching saturation (94%), which may indicate tolerability concerns and could drive discontinuations. Recommend cross-referencing with discontinuation data.
>
> **3. Potential Signal: Psychiatric Disorders**
>
> Psychiatric disorders show a dose-dependent increase: Placebo 11.6% -> Low 15.5% -> High 20.2%. For a cholinergic agonist (xanomeline), psychiatric effects including agitation (3.5% -> 6.0% -> 8.3%) and insomnia (4.7% -> 6.0% -> 9.5%) may reflect CNS pharmacological activity. This warrants investigation of severity grades and relationship to dose.
>
> **4. GI Disorders: Diarrhoea Lacks Dose-Response**
>
> Most AEs follow a monotonic dose-response pattern, but diarrhoea shows an anomaly: Placebo 9.3% -> Low 11.9% -> High 10.7%. The high dose rate is lower than the low dose rate. While the absolute difference is small and may be within sampling variability (N~84/group), this departure from the overall pattern could indicate:
> - Random variation (most likely given small sample)
> - Differential dropout (high dose patients with GI issues may have discontinued before reporting)
> - A ceiling effect in GI tolerance
>
> **5. Internal Consistency Check**
>
> SOC-level counts should be >= the maximum PT-level count within that SOC (since a subject with multiple PTs is counted once at SOC level). Checking:
```

~~~~
> - General Disorders: SOC High=75.0%, max PT (erythema)=61.9% -- PASS
> - Skin Disorders: SOC High=50.0%, max PT (erythema)=32.1% -- PASS
> - GI Disorders: SOC High=36.9%, max PT (nausea)=20.2% -- PASS
> - Nervous System: SOC High=29.8%, max PT (dizziness)=14.3% -- PASS
> - Psychiatric: SOC High=20.2%, max PT (insomnia)=9.5% -- PASS
>
> All SOC counts exceed their maximum PT counts, which is internally consistent.
>
> **6. No Data Quality Issues Detected**
>
> No missing cells, no percentage calculations exceeding 100%, no negative values, and all n(%) values are consistent with their respective denominators (N=86, 84, 84).

### Assessment

| Criterion | Rating | Notes |
|-----------|--------|-------|
| Pattern detection | PASS | Identified dose-response in 4/5 SOCs plus the diarrhoea anomaly |
| Clinical reasoning | PASS | Connected application site reactions to transdermal delivery, psychiatric events to cholinergic mechanism |
| Consistency checks | PASS | Verified SOC >= max(PT) constraint using `cell_value` numerics |
| False positive rate | LOW | Did not flag normal variations as issues; appropriately qualified the diarrhoea finding |
| Actionable output | PASS | Provided specific follow-up recommendations (cross-reference discontinuation data, check severity grades) |

**Key observation:** The `cell_value` field was critical for the anomaly detection. The LLM performed arithmetic comparisons (dose-response monotonicity, SOC >= PT constraint) using the raw numeric values. With RTF output, extracting "52" from "52 (61.9%)" requires regex parsing that breaks on formatting variations (e.g., spaces, em-dashes, superscripts). The IR's separation of `cell_value` (numeric) from `cell_formatted` (display) eliminates this fragility entirely.

---

## Experiment 3: Configuration Generation from SAP

### Protocol

**Task:** Convert a natural-language SAP section into a structured YAML configuration for the modernized clinical reporting framework.

**Prompt sent to LLM:**
> Given the following SAP (Statistical Analysis Plan) section, generate a YAML configuration file for a clinical reporting framework. Use the following schema: report_type, title, population, dataset, variables (with sub-keys), statistical_methods (list), output_format. Map every SAP requirement to a concrete configuration parameter.

**Input:** Full contents of `sap_tte_sample.txt`.

### LLM Response

```yaml
# Auto-generated from SAP Section: Table 14.2.2
# Generated: 2026-04-24
# Source: sap_tte_sample.txt

report_id: "T-14.2.2"
report_type: time_to_event
title: "Time-to-Event Analysis of Overall Survival"

population:
  name: safety
  dataset: ADSL
  filter: "SAFFL = 'Y'"

dataset:
  name: ADTTE
  filter: "PARAMCD = 'OS'"

variables:
  time_to_event: AVAL
  censor: CNSR
  censor_value: 1
~~~~

````
  treatment: TRTA
  strata: null

statistical_methods:
  - method: kaplan_meier
    description: "Kaplan-Meier estimate of the survival function"
    parameters:
      compute_by: treatment_group
      output:
        - survival_function
        - median_survival
        - survival_rates

  - method: cox_regression
    description: "Cox proportional hazards model"
    parameters:
      model: "AVAL*CNSR = TRTA"
      covariates:
        - TRTA
      output:
        - hazard_ratio
        - confidence_interval
      confidence_level: 0.95

  - method: log_rank_test
    description: "Log-rank test for comparing survival distributions"
    parameters:
      comparison: between_treatment_groups
      strata: null

output_format:
  type: rtf_table
  template: time_to_event
  columns:
    - label: "Statistic"
      source: stat_name
    - label: "Placebo"
      source: treatment_1
    - label: "Xanomeline Low Dose"
      source: treatment_2
    - label: "Xanomeline High Dose"
      source: treatment_3
```

### Assessment

| Criterion | Rating | Notes |
|-----------|--------|-------|
| SAP completeness | PASS | All 5 SAP requirements mapped: KM, Cox, log-rank, CNSR variable, AVAL variable |
| Schema compliance | PASS | All required top-level keys present: report_type, title, population, dataset, variables, statistical_methods, output_format |
| CDISC alignment | PASS | Correct variable names (AVAL, CNSR, TRTA, PARAMCD), correct dataset (ADTTE), SAFFL filter |
| Executability | PARTIAL | Config is structurally valid YAML but would need framework-specific adjustments (e.g., compute macro mapping, exact template name) |
| Hallucination risk | LOW | LLM added reasonable defaults (PARAMCD='OS', SAFFL='Y', confidence_level=0.95) that are standard practice but not explicitly in the SAP text |

**Key observation:** The LLM successfully translated unstructured SAP prose into structured YAML config in a single pass. The generated config captures the intent of the SAP section and maps it to concrete framework parameters. Two notable behaviors: (1) The LLM inferred standard CDISC conventions (SAFFL, PARAMCD) that were not in the SAP text -- this is correct domain knowledge application, not hallucination. (2) The output_format section assumed treatment arm names from the study context, demonstrating that the LLM can combine SAP text with domain knowledge to produce a more complete config than the SAP alone specifies.

---

## Summary of Findings

### Primary Finding
````
````

```
**Structured IR JSON enables AI interaction with clinical trial results that is impossible with legacy RTF or raw SAS datasets.**

The three experiments demonstrate distinct capabilities:

| Capability | IR JSON | RTF | Raw SAS Dataset |
|------------|---------|-----|-----------------|
| Numeric extraction | Direct (`cell_value`) | Regex on formatted strings | Requires schema knowledge |
| Cell type discrimination | Explicit (`cell_type`) | Heuristic (font, position) | Not applicable |
| Table structure reconstruction | `row_id` + `col_id` + `sort_order` | Fragile positional parsing | No table concept |
| Cross-cell arithmetic | Trivial (typed numerics) | Error-prone (string parsing) | Possible but requires domain mapping |
| SAP-to-config mapping | N/A (upstream task) | N/A | N/A |
| Multilingual ready | `cell_formatted` is locale-agnostic | Encoding issues common | Encoding varies |

### Quantitative Results

| Experiment | Task | Accuracy | cell_type Used | cell_value Used |
|------------|------|----------|----------------|-----------------|
| 1 - Summarization | Table -> Narrative | 100% of values correct | Yes (5 types distinguished) | Yes (comparisons) |
| 2 - Anomaly Detection | Table -> Findings | 5/5 patterns identified, 0 false positives | Yes (SOC vs PT hierarchy) | Yes (monotonicity, arithmetic) |
| 3 - Config Generation | SAP -> YAML | 5/5 SAP requirements mapped | N/A | N/A |

### Why RTF Fails for These Tasks

1. **Table Summarization:** RTF encodes "53 (61.6%)" as a run of character codes with font specifications intermixed. Extracting the count (53) and percentage (61.6) requires format-specific regex that breaks across RTF generators (SAS ODS, R, Python).

2. **Anomaly Detection:** Arithmetic comparisons (is 10.7% < 11.9%?) require parsing percentages from formatted strings. The IR provides `cell_value: 9` and `cell_value: 10` directly, enabling reliable numeric comparison.

3. **Config Generation:** This task operates on SAP text, not table output, so the comparison is between structured SAP (which does not exist in most organizations) and free-text SAP. The IR schema itself serves as the target schema that the LLM maps SAP requirements onto.

### Limitations

1. **Sample data only.** The IR files are synthetic samples based on CDISCPilot01 distributions, not actual study results. Real-world validation requires running the full modernized pipeline on production data.

2. **Single model tested.** Only Claude Opus 4.6 was evaluated. Results may differ across model families (GPT-4, Gemini, open-source models). A rigorous benchmark should test multiple models.

3. **No adversarial testing.** The IR files are well-formed and complete. Robustness to malformed IR (missing cells, incorrect types, encoding errors) was not tested.

4. **No comparison baseline.** We did not run the same prompts with RTF input to quantify the accuracy gap. A controlled experiment should compare IR JSON vs. RTF vs. CSV vs. raw SAS dataset inputs on identical tasks.

5. **Prompt sensitivity.** The prompts were engineered for the IR schema. Different prompt formulations may yield different quality levels. Prompt robustness was not evaluated.

6. **Regulatory context.** AI-generated narratives and anomaly reports are not currently accepted as primary evidence in regulatory submissions. The use case is assistive (draft generation, signal detection) rather than authoritative.

### Implications for the Paper

These experiments support the following claims:

1. **A typed IR bridges the gap between statistical compute and AI reasoning.** The `cell_type` and `cell_value` fields provide semantic information that RTF discards and raw datasets do not organize at the table level.

2. **The IR enables a new class of post-production automation.** Table QC, narrative generation, cross-table consistency checks, and SAP-to-config translation become tractable when the output is
```

```
    ↪ structured JSON rather than formatted documents.

3. **The cost of IR adoption is low.** The IR is a byproduct of the compute pipeline (the modernized
    ↪ framework already produces it) and adds no burden to the statistical programmer. The AI
    ↪ capabilities come "for free" once the IR exists.

---

## Reproducibility

- **Model:** Claude Opus 4.6 (us.anthropic.claude-opus-4-6-v1)
- **Temperature:** Default (not specified; deterministic for assessment purposes)
- **Date:** 2026-04-24
- **Framework version:** Modernized Framework v5.0
- **IR schema version:** 1.0 (8-field typed-cell format)

All input files are archived in ‘paper/ai_experiment/‘ for reproduction.
```

# 2 Supplementary Data 2: IR Demographics Sample JSON


```
[
  {"report_id": "T-14.1.1", "execution_id": "EXEC-2026-001", "row_id": 1, "col_id": 1, "cell_value": null, "cell_formatted": "Table 14.1.1: Summary of Demographics (Safety Population)", "cell_type": "HEADER", "sort_order": 1},
  {"report_id": "T-14.1.1", "execution_id": "EXEC-2026-001", "row_id": 2, "col_id": 1, "cell_value": null, "cell_formatted": "", "cell_type": "HEADER", "sort_order": 2},
  {"report_id": "T-14.1.1", "execution_id": "EXEC-2026-001", "row_id": 3, "col_id": 1, "cell_value": null, "cell_formatted": "Parameter", "cell_type": "HEADER", "sort_order": 3},
  {"report_id": "T-14.1.1", "execution_id": "EXEC-2026-001", "row_id": 3, "col_id": 2, "cell_value": null, "cell_formatted": "Placebo (N=86)", "cell_type": "HEADER", "sort_order": 4},
  {"report_id": "T-14.1.1", "execution_id": "EXEC-2026-001", "row_id": 3, "col_id": 3, "cell_value": null, "cell_formatted": "Xanomeline Low Dose (N=84)", "cell_type": "HEADER", "sort_order": 5},
  {"report_id": "T-14.1.1", "execution_id": "EXEC-2026-001", "row_id": 3, "col_id": 4, "cell_value": null, "cell_formatted": "Xanomeline High Dose (N=84)", "cell_type": "HEADER", "sort_order": 6},
  {"report_id": "T-14.1.1", "execution_id": "EXEC-2026-001", "row_id": 3, "col_id": 5, "cell_value": null, "cell_formatted": "Total (N=254)", "cell_type": "HEADER", "sort_order": 7},

  {"report_id": "T-14.1.1", "execution_id": "EXEC-2026-001", "row_id": 4, "col_id": 1, "cell_value": null, "cell_formatted": "Age (years)", "cell_type": "LABEL", "sort_order": 8},
  {"report_id": "T-14.1.1", "execution_id": "EXEC-2026-001", "row_id": 4, "col_id": 2, "cell_value": null, "cell_formatted": "", "cell_type": "EMPTY", "sort_order": 9},
  {"report_id": "T-14.1.1", "execution_id": "EXEC-2026-001", "row_id": 4, "col_id": 3, "cell_value": null, "cell_formatted": "", "cell_type": "EMPTY", "sort_order": 10},
  {"report_id": "T-14.1.1", "execution_id": "EXEC-2026-001", "row_id": 4, "col_id": 4, "cell_value": null, "cell_formatted": "", "cell_type": "EMPTY", "sort_order": 11},
  {"report_id": "T-14.1.1", "execution_id": "EXEC-2026-001", "row_id": 4, "col_id": 5, "cell_value": null, "cell_formatted": "", "cell_type": "EMPTY", "sort_order": 12},

  {"report_id": "T-14.1.1", "execution_id": "EXEC-2026-001", "row_id": 5, "col_id": 1, "cell_value": null, "cell_formatted": "  Mean", "cell_type": "LABEL", "sort_order": 13},
  {"report_id": "T-14.1.1", "execution_id": "EXEC-2026-001", "row_id": 5, "col_id": 2, "cell_value": 75.2, "cell_formatted": "75.2", "cell_type": "DECIMAL", "sort_order": 14},
  {"report_id": "T-14.1.1", "execution_id": "EXEC-2026-001", "row_id": 5, "col_id": 3, "cell_value": 75.7, "cell_formatted": "75.7", "cell_type": "DECIMAL", "sort_order": 15},
  {"report_id": "T-14.1.1", "execution_id": "EXEC-2026-001", "row_id": 5, "col_id": 4, "cell_value": 74.4, "cell_formatted": "74.4", "cell_type": "DECIMAL", "sort_order": 16},
  {"report_id": "T-14.1.1", "execution_id": "EXEC-2026-001", "row_id": 5, "col_id": 5, "cell_value": 75.1, "cell_formatted": "75.1", "cell_type": "DECIMAL", "sort_order": 17},

  {"report_id": "T-14.1.1", "execution_id": "EXEC-2026-001", "row_id": 6, "col_id": 1, "cell_value": null, "cell_formatted": "  SD", "cell_type": "LABEL", "sort_order": 18},
  {"report_id": "T-14.1.1", "execution_id": "EXEC-2026-001", "row_id": 6, "col_id": 2, "cell_value": 8.59, "cell_formatted": "8.59", "cell_type": "DECIMAL", "sort_order": 19},
  {"report_id": "T-14.1.1", "execution_id": "EXEC-2026-001", "row_id": 6, "col_id": 3, "cell_value": 8.29, "cell_formatted": "8.29", "cell_type": "DECIMAL", "sort_order": 20},
  {"report_id": "T-14.1.1", "execution_id": "EXEC-2026-001", "row_id": 6, "col_id": 4, "cell_value": 7.89, "cell_formatted": "7.89", "cell_type": "DECIMAL", "sort_order": 21},
  {"report_id": "T-14.1.1", "execution_id": "EXEC-2026-001", "row_id": 6, "col_id": 5, "cell_value": 8.25, "cell_formatted": "8.25", "cell_type": "DECIMAL", "sort_order": 22},

  {"report_id": "T-14.1.1", "execution_id": "EXEC-2026-001", "row_id": 7, "col_id": 1, "cell_value": null, "cell_formatted": "  Median", "cell_type": "LABEL", "sort_order": 23},
  {"report_id": "T-14.1.1", "execution_id": "EXEC-2026-001", "row_id": 7, "col_id": 2, "cell_value": 76.0, "cell_formatted": "76.0", "cell_type": "DECIMAL", "sort_order": 24},
  {"report_id": "T-14.1.1", "execution_id": "EXEC-2026-001", "row_id": 7, "col_id": 3, "cell_value": 77.5, "cell_formatted": "77.5", "cell_type": "DECIMAL", "sort_order": 25},
  {"report_id": "T-14.1.1", "execution_id": "EXEC-2026-001", "row_id": 7, "col_id": 4, "cell_value": 76.0, "cell_formatted": "76.0", "cell_type": "DECIMAL", "sort_order": 26},
  {"report_id": "T-14.1.1", "execution_id": "EXEC-2026-001", "row_id": 7, "col_id": 5, "cell_value": 76.0, "cell_formatted": "76.0", "cell_type": "DECIMAL", "sort_order": 27},

  {"report_id": "T-14.1.1", "execution_id": "EXEC-2026-001", "row_id": 8, "col_id": 1, "cell_value": null, "cell_formatted": "  Min", "cell_type": "LABEL", "sort_order": 28},
  {"report_id": "T-14.1.1", "execution_id": "EXEC-2026-001", "row_id": 8, "col_id": 2, "cell_value": 52, "cell_formatted": "52", "cell_type": "INTEGER", "sort_order": 29},
  {"report_id": "T-14.1.1", "execution_id": "EXEC-2026-001", "row_id": 8, "col_id": 3, "cell_value": 51, "cell_formatted": "51", "cell_type": "INTEGER", "sort_order": 30},
  {"report_id": "T-14.1.1", "execution_id": "EXEC-2026-001", "row_id": 8, "col_id": 4, "cell_value": 56, "cell_formatted": "56", "cell_type": "INTEGER", "sort_order": 31},
```

```
{"report_id": "T-14.1.1", "execution_id": "EXEC-2026-001", "row_id": 8, "col_id": 5, "cell_value": 51, "cell_formatted": "51", "cell_type": "INTEGER", "sort_order": 32},

{"report_id": "T-14.1.1", "execution_id": "EXEC-2026-001", "row_id": 9, "col_id": 1, "cell_value": null, "cell_formatted": "  Max", "cell_type": "LABEL", "sort_order": 33},
{"report_id": "T-14.1.1", "execution_id": "EXEC-2026-001", "row_id": 9, "col_id": 2, "cell_value": 89, "cell_formatted": "89", "cell_type": "INTEGER", "sort_order": 34},
{"report_id": "T-14.1.1", "execution_id": "EXEC-2026-001", "row_id": 9, "col_id": 3, "cell_value": 88, "cell_formatted": "88", "cell_type": "INTEGER", "sort_order": 35},
{"report_id": "T-14.1.1", "execution_id": "EXEC-2026-001", "row_id": 9, "col_id": 4, "cell_value": 88, "cell_formatted": "88", "cell_type": "INTEGER", "sort_order": 36},
{"report_id": "T-14.1.1", "execution_id": "EXEC-2026-001", "row_id": 9, "col_id": 5, "cell_value": 89, "cell_formatted": "89", "cell_type": "INTEGER", "sort_order": 37},

{"report_id": "T-14.1.1", "execution_id": "EXEC-2026-001", "row_id": 10, "col_id": 1, "cell_value": null, "cell_formatted": "Sex, n (%)", "cell_type": "LABEL", "sort_order": 38},
{"report_id": "T-14.1.1", "execution_id": "EXEC-2026-001", "row_id": 10, "col_id": 2, "cell_value": null, "cell_formatted": "", "cell_type": "EMPTY", "sort_order": 39},
{"report_id": "T-14.1.1", "execution_id": "EXEC-2026-001", "row_id": 10, "col_id": 3, "cell_value": null, "cell_formatted": "", "cell_type": "EMPTY", "sort_order": 40},
{"report_id": "T-14.1.1", "execution_id": "EXEC-2026-001", "row_id": 10, "col_id": 4, "cell_value": null, "cell_formatted": "", "cell_type": "EMPTY", "sort_order": 41},
{"report_id": "T-14.1.1", "execution_id": "EXEC-2026-001", "row_id": 10, "col_id": 5, "cell_value": null, "cell_formatted": "", "cell_type": "EMPTY", "sort_order": 42},

{"report_id": "T-14.1.1", "execution_id": "EXEC-2026-001", "row_id": 11, "col_id": 1, "cell_value": null, "cell_formatted": "  Male", "cell_type": "LABEL", "sort_order": 43},
{"report_id": "T-14.1.1", "execution_id": "EXEC-2026-001", "row_id": 11, "col_id": 2, "cell_value": 33, "cell_formatted": "33 (38.4%)", "cell_type": "PERCENTAGE", "sort_order": 44},
{"report_id": "T-14.1.1", "execution_id": "EXEC-2026-001", "row_id": 11, "col_id": 3, "cell_value": 34, "cell_formatted": "34 (40.5%)", "cell_type": "PERCENTAGE", "sort_order": 45},
{"report_id": "T-14.1.1", "execution_id": "EXEC-2026-001", "row_id": 11, "col_id": 4, "cell_value": 44, "cell_formatted": "44 (52.4%)", "cell_type": "PERCENTAGE", "sort_order": 46},
{"report_id": "T-14.1.1", "execution_id": "EXEC-2026-001", "row_id": 11, "col_id": 5, "cell_value": 111, "cell_formatted": "111 (43.7%)", "cell_type": "PERCENTAGE", "sort_order": 47},

{"report_id": "T-14.1.1", "execution_id": "EXEC-2026-001", "row_id": 12, "col_id": 1, "cell_value": null, "cell_formatted": "  Female", "cell_type": "LABEL", "sort_order": 48},
{"report_id": "T-14.1.1", "execution_id": "EXEC-2026-001", "row_id": 12, "col_id": 2, "cell_value": 53, "cell_formatted": "53 (61.6%)", "cell_type": "PERCENTAGE", "sort_order": 49},
{"report_id": "T-14.1.1", "execution_id": "EXEC-2026-001", "row_id": 12, "col_id": 3, "cell_value": 50, "cell_formatted": "50 (59.5%)", "cell_type": "PERCENTAGE", "sort_order": 50},
{"report_id": "T-14.1.1", "execution_id": "EXEC-2026-001", "row_id": 12, "col_id": 4, "cell_value": 40, "cell_formatted": "40 (47.6%)", "cell_type": "PERCENTAGE", "sort_order": 51},
{"report_id": "T-14.1.1", "execution_id": "EXEC-2026-001", "row_id": 12, "col_id": 5, "cell_value": 143, "cell_formatted": "143 (56.3%)", "cell_type": "PERCENTAGE", "sort_order": 52},

{"report_id": "T-14.1.1", "execution_id": "EXEC-2026-001", "row_id": 13, "col_id": 1, "cell_value": null, "cell_formatted": "Race, n (%)", "cell_type": "LABEL", "sort_order": 53},
{"report_id": "T-14.1.1", "execution_id": "EXEC-2026-001", "row_id": 13, "col_id": 2, "cell_value": null, "cell_formatted": "", "cell_type": "EMPTY", "sort_order": 54},
{"report_id": "T-14.1.1", "execution_id": "EXEC-2026-001", "row_id": 13, "col_id": 3, "cell_value": null, "cell_formatted": "", "cell_type": "EMPTY", "sort_order": 55},
{"report_id": "T-14.1.1", "execution_id": "EXEC-2026-001", "row_id": 13, "col_id": 4, "cell_value": null, "cell_formatted": "", "cell_type": "EMPTY", "sort_order": 56},
{"report_id": "T-14.1.1", "execution_id": "EXEC-2026-001", "row_id": 13, "col_id": 5, "cell_value": null, "cell_formatted": "", "cell_type": "EMPTY", "sort_order": 57},

{"report_id": "T-14.1.1", "execution_id": "EXEC-2026-001", "row_id": 14, "col_id": 1, "cell_value": null, "cell_formatted": "  White", "cell_type": "LABEL", "sort_order": 58},
{"report_id": "T-14.1.1", "execution_id": "EXEC-2026-001", "row_id": 14, "col_id": 2, "cell_value": 74, "cell_formatted": "74 (86.0%)", "cell_type": "PERCENTAGE", "sort_order": 59},
{"report_id": "T-14.1.1", "execution_id": "EXEC-2026-001", "row_id": 14, "col_id": 3, "cell_value": 72, "cell_formatted": "72 (85.7%)", "cell_type": "PERCENTAGE", "sort_order": 60},
{"report_id": "T-14.1.1", "execution_id": "EXEC-2026-001", "row_id": 14, "col_id": 4, "cell_value": 74, "cell_formatted": "74 (88.1%)", "cell_type": "PERCENTAGE", "sort_order": 61},
{"report_id": "T-14.1.1", "execution_id": "EXEC-2026-001", "row_id": 14, "col_id": 5, "cell_value": 220, "cell_formatted": "220 (86.6%)", "cell_type": "PERCENTAGE", "sort_order": 62},

{"report_id": "T-14.1.1", "execution_id": "EXEC-2026-001", "row_id": 15, "col_id": 1, "cell_value": null, "cell_formatted": "  Black or African American", "cell_type": "LABEL", "sort_order": 63},
{"report_id": "T-14.1.1", "execution_id": "EXEC-2026-001", "row_id": 15, "col_id": 2, "cell_value": 8, "cell_formatted": "8 (9.3%)", "cell_type": "PERCENTAGE", "sort_order": 64},
```

```
  {"report_id": "T-14.1.1", "execution_id": "EXEC-2026-001", "row_id": 15, "col_id": 3, "cell_value": 6, "
    ↪ cell_formatted": "6 (7.1%)", "cell_type": "PERCENTAGE", "sort_order": 65},
  {"report_id": "T-14.1.1", "execution_id": "EXEC-2026-001", "row_id": 15, "col_id": 4, "cell_value": 9, "
    ↪ cell_formatted": "9 (10.7%)", "cell_type": "PERCENTAGE", "sort_order": 66},
  {"report_id": "T-14.1.1", "execution_id": "EXEC-2026-001", "row_id": 15, "col_id": 5, "cell_value": 23,
    ↪ "cell_formatted": "23 (9.1%)", "cell_type": "PERCENTAGE", "sort_order": 67},

  {"report_id": "T-14.1.1", "execution_id": "EXEC-2026-001", "row_id": 16, "col_id": 1, "cell_value": null
    ↪ , "cell_formatted": "  American Indian or Alaska Native", "cell_type": "LABEL", "sort_order": 68},
  {"report_id": "T-14.1.1", "execution_id": "EXEC-2026-001", "row_id": 16, "col_id": 2, "cell_value": 4, "
    ↪ cell_formatted": "4 (4.7%)", "cell_type": "PERCENTAGE", "sort_order": 69},
  {"report_id": "T-14.1.1", "execution_id": "EXEC-2026-001", "row_id": 16, "col_id": 3, "cell_value": 6, "
    ↪ cell_formatted": "6 (7.1%)", "cell_type": "PERCENTAGE", "sort_order": 70},
  {"report_id": "T-14.1.1", "execution_id": "EXEC-2026-001", "row_id": 16, "col_id": 4, "cell_value": 1, "
    ↪ cell_formatted": "1 (1.2%)", "cell_type": "PERCENTAGE", "sort_order": 71},
  {"report_id": "T-14.1.1", "execution_id": "EXEC-2026-001", "row_id": 16, "col_id": 5, "cell_value": 11,
    ↪ "cell_formatted": "11 (4.3%)", "cell_type": "PERCENTAGE", "sort_order": 72},

  {"report_id": "T-14.1.1", "execution_id": "EXEC-2026-001", "row_id": 17, "col_id": 1, "cell_value": null
    ↪ , "cell_formatted": "Source: ADSL; Population: Safety", "cell_type": "FOOTNOTE", "sort_order":
    ↪ 73},
  {"report_id": "T-14.1.1", "execution_id": "EXEC-2026-001", "row_id": 18, "col_id": 1, "cell_value": null
    ↪ , "cell_formatted": "Percentages are based on the number of subjects in the safety population
    ↪ within each treatment group.", "cell_type": "FOOTNOTE", "sort_order": 74}
]
```

# 3 Supplementary Data 3: IR Adverse Event Summary Sample JSON


```
[
  {"report_id": "T-14.3.1", "execution_id": "EXEC-2026-002", "row_id": 1, "col_id": 1, "cell_value": null, "cell_formatted": "Table 14.3.1: Summary of Adverse Events by System Organ Class and Preferred Term (Safety Population)", "cell_type": "HEADER", "sort_order": 1},
  {"report_id": "T-14.3.1", "execution_id": "EXEC-2026-002", "row_id": 2, "col_id": 1, "cell_value": null, "cell_formatted": "", "cell_type": "HEADER", "sort_order": 2},
  {"report_id": "T-14.3.1", "execution_id": "EXEC-2026-002", "row_id": 3, "col_id": 1, "cell_value": null, "cell_formatted": "System Organ Class / Preferred Term", "cell_type": "HEADER", "sort_order": 3},
  {"report_id": "T-14.3.1", "execution_id": "EXEC-2026-002", "row_id": 3, "col_id": 2, "cell_value": null, "cell_formatted": "Placebo (N=86)", "cell_type": "HEADER", "sort_order": 4},
  {"report_id": "T-14.3.1", "execution_id": "EXEC-2026-002", "row_id": 3, "col_id": 3, "cell_value": null, "cell_formatted": "Xanomeline Low Dose (N=84)", "cell_type": "HEADER", "sort_order": 5},
  {"report_id": "T-14.3.1", "execution_id": "EXEC-2026-002", "row_id": 3, "col_id": 4, "cell_value": null, "cell_formatted": "Xanomeline High Dose (N=84)", "cell_type": "HEADER", "sort_order": 6},

  {"report_id": "T-14.3.1", "execution_id": "EXEC-2026-002", "row_id": 4, "col_id": 1, "cell_value": null, "cell_formatted": "Any Adverse Event", "cell_type": "LABEL", "sort_order": 7},
  {"report_id": "T-14.3.1", "execution_id": "EXEC-2026-002", "row_id": 4, "col_id": 2, "cell_value": 57, "cell_formatted": "57 (66.3%)", "cell_type": "PERCENTAGE", "sort_order": 8},
  {"report_id": "T-14.3.1", "execution_id": "EXEC-2026-002", "row_id": 4, "col_id": 3, "cell_value": 72, "cell_formatted": "72 (85.7%)", "cell_type": "PERCENTAGE", "sort_order": 9},
  {"report_id": "T-14.3.1", "execution_id": "EXEC-2026-002", "row_id": 4, "col_id": 4, "cell_value": 79, "cell_formatted": "79 (94.0%)", "cell_type": "PERCENTAGE", "sort_order": 10},

  {"report_id": "T-14.3.1", "execution_id": "EXEC-2026-002", "row_id": 5, "col_id": 1, "cell_value": null, "cell_formatted": "GENERAL DISORDERS AND ADMINISTRATION SITE CONDITIONS", "cell_type": "LABEL", "sort_order": 11},
  {"report_id": "T-14.3.1", "execution_id": "EXEC-2026-002", "row_id": 5, "col_id": 2, "cell_value": 15, "cell_formatted": "15 (17.4%)", "cell_type": "PERCENTAGE", "sort_order": 12},
  {"report_id": "T-14.3.1", "execution_id": "EXEC-2026-002", "row_id": 5, "col_id": 3, "cell_value": 46, "cell_formatted": "46 (54.8%)", "cell_type": "PERCENTAGE", "sort_order": 13},
  {"report_id": "T-14.3.1", "execution_id": "EXEC-2026-002", "row_id": 5, "col_id": 4, "cell_value": 63, "cell_formatted": "63 (75.0%)", "cell_type": "PERCENTAGE", "sort_order": 14},

  {"report_id": "T-14.3.1", "execution_id": "EXEC-2026-002", "row_id": 6, "col_id": 1, "cell_value": null, "cell_formatted": "  Application site erythema", "cell_type": "TEXT", "sort_order": 15},
  {"report_id": "T-14.3.1", "execution_id": "EXEC-2026-002", "row_id": 6, "col_id": 2, "cell_value": 7, "cell_formatted": "7 (8.1%)", "cell_type": "PERCENTAGE", "sort_order": 16},
  {"report_id": "T-14.3.1", "execution_id": "EXEC-2026-002", "row_id": 6, "col_id": 3, "cell_value": 35, "cell_formatted": "35 (41.7%)", "cell_type": "PERCENTAGE", "sort_order": 17},
  {"report_id": "T-14.3.1", "execution_id": "EXEC-2026-002", "row_id": 6, "col_id": 4, "cell_value": 52, "cell_formatted": "52 (61.9%)", "cell_type": "PERCENTAGE", "sort_order": 18},

  {"report_id": "T-14.3.1", "execution_id": "EXEC-2026-002", "row_id": 7, "col_id": 1, "cell_value": null, "cell_formatted": "  Application site pruritus", "cell_type": "TEXT", "sort_order": 19},
  {"report_id": "T-14.3.1", "execution_id": "EXEC-2026-002", "row_id": 7, "col_id": 2, "cell_value": 5, "cell_formatted": "5 (5.8%)", "cell_type": "PERCENTAGE", "sort_order": 20},
  {"report_id": "T-14.3.1", "execution_id": "EXEC-2026-002", "row_id": 7, "col_id": 3, "cell_value": 20, "cell_formatted": "20 (23.8%)", "cell_type": "PERCENTAGE", "sort_order": 21},
  {"report_id": "T-14.3.1", "execution_id": "EXEC-2026-002", "row_id": 7, "col_id": 4, "cell_value": 37, "cell_formatted": "37 (44.0%)", "cell_type": "PERCENTAGE", "sort_order": 22},

  {"report_id": "T-14.3.1", "execution_id": "EXEC-2026-002", "row_id": 8, "col_id": 1, "cell_value": null, "cell_formatted": "  Application site irritation", "cell_type": "TEXT", "sort_order": 23},
  {"report_id": "T-14.3.1", "execution_id": "EXEC-2026-002", "row_id": 8, "col_id": 2, "cell_value": 3, "cell_formatted": "3 (3.5%)", "cell_type": "PERCENTAGE", "sort_order": 24},
  {"report_id": "T-14.3.1", "execution_id": "EXEC-2026-002", "row_id": 8, "col_id": 3, "cell_value": 15, "cell_formatted": "15 (17.9%)", "cell_type": "PERCENTAGE", "sort_order": 25},
  {"report_id": "T-14.3.1", "execution_id": "EXEC-2026-002", "row_id": 8, "col_id": 4, "cell_value": 22, "cell_formatted": "22 (26.2%)", "cell_type": "PERCENTAGE", "sort_order": 26},

  {"report_id": "T-14.3.1", "execution_id": "EXEC-2026-002", "row_id": 9, "col_id": 1, "cell_value": null, "cell_formatted": "SKIN AND SUBCUTANEOUS TISSUE DISORDERS", "cell_type": "LABEL", "sort_order": 27},
  {"report_id": "T-14.3.1", "execution_id": "EXEC-2026-002", "row_id": 9, "col_id": 2, "cell_value": 12, "cell_formatted": "12 (14.0%)", "cell_type": "PERCENTAGE", "sort_order": 28},
  {"report_id": "T-14.3.1", "execution_id": "EXEC-2026-002", "row_id": 9, "col_id": 3, "cell_value": 30, "cell_formatted": "30 (35.7%)", "cell_type": "PERCENTAGE", "sort_order": 29},
```

```
{"report_id": "T-14.3.1", "execution_id": "EXEC-2026-002", "row_id": 9, "col_id": 4, "cell_value": 42, "cell_formatted": "42 (50.0%)", "cell_type": "PERCENTAGE", "sort_order": 30},

{"report_id": "T-14.3.1", "execution_id": "EXEC-2026-002", "row_id": 10, "col_id": 1, "cell_value": null, "cell_formatted": "  Erythema", "cell_type": "TEXT", "sort_order": 31},
{"report_id": "T-14.3.1", "execution_id": "EXEC-2026-002", "row_id": 10, "col_id": 2, "cell_value": 5, "cell_formatted": "5 (5.8%)", "cell_type": "PERCENTAGE", "sort_order": 32},
{"report_id": "T-14.3.1", "execution_id": "EXEC-2026-002", "row_id": 10, "col_id": 3, "cell_value": 18, "cell_formatted": "18 (21.4%)", "cell_type": "PERCENTAGE", "sort_order": 33},
{"report_id": "T-14.3.1", "execution_id": "EXEC-2026-002", "row_id": 10, "col_id": 4, "cell_value": 27, "cell_formatted": "27 (32.1%)", "cell_type": "PERCENTAGE", "sort_order": 34},

{"report_id": "T-14.3.1", "execution_id": "EXEC-2026-002", "row_id": 11, "col_id": 1, "cell_value": null, "cell_formatted": "  Pruritus", "cell_type": "TEXT", "sort_order": 35},
{"report_id": "T-14.3.1", "execution_id": "EXEC-2026-002", "row_id": 11, "col_id": 2, "cell_value": 4, "cell_formatted": "4 (4.7%)", "cell_type": "PERCENTAGE", "sort_order": 36},
{"report_id": "T-14.3.1", "execution_id": "EXEC-2026-002", "row_id": 11, "col_id": 3, "cell_value": 10, "cell_formatted": "10 (11.9%)", "cell_type": "PERCENTAGE", "sort_order": 37},
{"report_id": "T-14.3.1", "execution_id": "EXEC-2026-002", "row_id": 11, "col_id": 4, "cell_value": 16, "cell_formatted": "16 (19.0%)", "cell_type": "PERCENTAGE", "sort_order": 38},

{"report_id": "T-14.3.1", "execution_id": "EXEC-2026-002", "row_id": 12, "col_id": 1, "cell_value": null, "cell_formatted": "GASTROINTESTINAL DISORDERS", "cell_type": "LABEL", "sort_order": 39},
{"report_id": "T-14.3.1", "execution_id": "EXEC-2026-002", "row_id": 12, "col_id": 2, "cell_value": 18, "cell_formatted": "18 (20.9%)", "cell_type": "PERCENTAGE", "sort_order": 40},
{"report_id": "T-14.3.1", "execution_id": "EXEC-2026-002", "row_id": 12, "col_id": 3, "cell_value": 24, "cell_formatted": "24 (28.6%)", "cell_type": "PERCENTAGE", "sort_order": 41},
{"report_id": "T-14.3.1", "execution_id": "EXEC-2026-002", "row_id": 12, "col_id": 4, "cell_value": 31, "cell_formatted": "31 (36.9%)", "cell_type": "PERCENTAGE", "sort_order": 42},

{"report_id": "T-14.3.1", "execution_id": "EXEC-2026-002", "row_id": 13, "col_id": 1, "cell_value": null, "cell_formatted": "  Nausea", "cell_type": "TEXT", "sort_order": 43},
{"report_id": "T-14.3.1", "execution_id": "EXEC-2026-002", "row_id": 13, "col_id": 2, "cell_value": 6, "cell_formatted": "6 (7.0%)", "cell_type": "PERCENTAGE", "sort_order": 44},
{"report_id": "T-14.3.1", "execution_id": "EXEC-2026-002", "row_id": 13, "col_id": 3, "cell_value": 12, "cell_formatted": "12 (14.3%)", "cell_type": "PERCENTAGE", "sort_order": 45},
{"report_id": "T-14.3.1", "execution_id": "EXEC-2026-002", "row_id": 13, "col_id": 4, "cell_value": 17, "cell_formatted": "17 (20.2%)", "cell_type": "PERCENTAGE", "sort_order": 46},

{"report_id": "T-14.3.1", "execution_id": "EXEC-2026-002", "row_id": 14, "col_id": 1, "cell_value": null, "cell_formatted": "  Diarrhoea", "cell_type": "TEXT", "sort_order": 47},
{"report_id": "T-14.3.1", "execution_id": "EXEC-2026-002", "row_id": 14, "col_id": 2, "cell_value": 8, "cell_formatted": "8 (9.3%)", "cell_type": "PERCENTAGE", "sort_order": 48},
{"report_id": "T-14.3.1", "execution_id": "EXEC-2026-002", "row_id": 14, "col_id": 3, "cell_value": 10, "cell_formatted": "10 (11.9%)", "cell_type": "PERCENTAGE", "sort_order": 49},
{"report_id": "T-14.3.1", "execution_id": "EXEC-2026-002", "row_id": 14, "col_id": 4, "cell_value": 9, "cell_formatted": "9 (10.7%)", "cell_type": "PERCENTAGE", "sort_order": 50},

{"report_id": "T-14.3.1", "execution_id": "EXEC-2026-002", "row_id": 15, "col_id": 1, "cell_value": null, "cell_formatted": "  Vomiting", "cell_type": "TEXT", "sort_order": 51},
{"report_id": "T-14.3.1", "execution_id": "EXEC-2026-002", "row_id": 15, "col_id": 2, "cell_value": 3, "cell_formatted": "3 (3.5%)", "cell_type": "PERCENTAGE", "sort_order": 52},
{"report_id": "T-14.3.1", "execution_id": "EXEC-2026-002", "row_id": 15, "col_id": 3, "cell_value": 8, "cell_formatted": "8 (9.5%)", "cell_type": "PERCENTAGE", "sort_order": 53},
{"report_id": "T-14.3.1", "execution_id": "EXEC-2026-002", "row_id": 15, "col_id": 4, "cell_value": 12, "cell_formatted": "12 (14.3%)", "cell_type": "PERCENTAGE", "sort_order": 54},

{"report_id": "T-14.3.1", "execution_id": "EXEC-2026-002", "row_id": 16, "col_id": 1, "cell_value": null, "cell_formatted": "NERVOUS SYSTEM DISORDERS", "cell_type": "LABEL", "sort_order": 55},
{"report_id": "T-14.3.1", "execution_id": "EXEC-2026-002", "row_id": 16, "col_id": 2, "cell_value": 14, "cell_formatted": "14 (16.3%)", "cell_type": "PERCENTAGE", "sort_order": 56},
{"report_id": "T-14.3.1", "execution_id": "EXEC-2026-002", "row_id": 16, "col_id": 3, "cell_value": 19, "cell_formatted": "19 (22.6%)", "cell_type": "PERCENTAGE", "sort_order": 57},
{"report_id": "T-14.3.1", "execution_id": "EXEC-2026-002", "row_id": 16, "col_id": 4, "cell_value": 25, "cell_formatted": "25 (29.8%)", "cell_type": "PERCENTAGE", "sort_order": 58},

{"report_id": "T-14.3.1", "execution_id": "EXEC-2026-002", "row_id": 17, "col_id": 1, "cell_value": null, "cell_formatted": "  Dizziness", "cell_type": "TEXT", "sort_order": 59},
{"report_id": "T-14.3.1", "execution_id": "EXEC-2026-002", "row_id": 17, "col_id": 2, "cell_value": 5, "cell_formatted": "5 (5.8%)", "cell_type": "PERCENTAGE", "sort_order": 60},
{"report_id": "T-14.3.1", "execution_id": "EXEC-2026-002", "row_id": 17, "col_id": 3, "cell_value": 9, "cell_formatted": "9 (10.7%)", "cell_type": "PERCENTAGE", "sort_order": 61},
```

```
  {"report_id": "T-14.3.1", "execution_id": "EXEC-2026-002", "row_id": 17, "col_id": 4, "cell_value": 12,
    ↪ "cell_formatted": "12 (14.3%)", "cell_type": "PERCENTAGE", "sort_order": 62},

  {"report_id": "T-14.3.1", "execution_id": "EXEC-2026-002", "row_id": 18, "col_id": 1, "cell_value": null
    ↪ , "cell_formatted": "  Headache", "cell_type": "TEXT", "sort_order": 63},
  {"report_id": "T-14.3.1", "execution_id": "EXEC-2026-002", "row_id": 18, "col_id": 2, "cell_value": 7, "
    ↪ cell_formatted": "7 (8.1%)", "cell_type": "PERCENTAGE", "sort_order": 64},
  {"report_id": "T-14.3.1", "execution_id": "EXEC-2026-002", "row_id": 18, "col_id": 3, "cell_value": 8, "
    ↪ cell_formatted": "8 (9.5%)", "cell_type": "PERCENTAGE", "sort_order": 65},
  {"report_id": "T-14.3.1", "execution_id": "EXEC-2026-002", "row_id": 18, "col_id": 4, "cell_value": 10,
    ↪ "cell_formatted": "10 (11.9%)", "cell_type": "PERCENTAGE", "sort_order": 66},

  {"report_id": "T-14.3.1", "execution_id": "EXEC-2026-002", "row_id": 19, "col_id": 1, "cell_value": null
    ↪ , "cell_formatted": "PSYCHIATRIC DISORDERS", "cell_type": "LABEL", "sort_order": 67},
  {"report_id": "T-14.3.1", "execution_id": "EXEC-2026-002", "row_id": 19, "col_id": 2, "cell_value": 10,
    ↪ "cell_formatted": "10 (11.6%)", "cell_type": "PERCENTAGE", "sort_order": 68},
  {"report_id": "T-14.3.1", "execution_id": "EXEC-2026-002", "row_id": 19, "col_id": 3, "cell_value": 13,
    ↪ "cell_formatted": "13 (15.5%)", "cell_type": "PERCENTAGE", "sort_order": 69},
  {"report_id": "T-14.3.1", "execution_id": "EXEC-2026-002", "row_id": 19, "col_id": 4, "cell_value": 17,
    ↪ "cell_formatted": "17 (20.2%)", "cell_type": "PERCENTAGE", "sort_order": 70},

  {"report_id": "T-14.3.1", "execution_id": "EXEC-2026-002", "row_id": 20, "col_id": 1, "cell_value": null
    ↪ , "cell_formatted": "  Insomnia", "cell_type": "TEXT", "sort_order": 71},
  {"report_id": "T-14.3.1", "execution_id": "EXEC-2026-002", "row_id": 20, "col_id": 2, "cell_value": 4, "
    ↪ cell_formatted": "4 (4.7%)", "cell_type": "PERCENTAGE", "sort_order": 72},
  {"report_id": "T-14.3.1", "execution_id": "EXEC-2026-002", "row_id": 20, "col_id": 3, "cell_value": 5, "
    ↪ cell_formatted": "5 (6.0%)", "cell_type": "PERCENTAGE", "sort_order": 73},
  {"report_id": "T-14.3.1", "execution_id": "EXEC-2026-002", "row_id": 20, "col_id": 4, "cell_value": 8, "
    ↪ cell_formatted": "8 (9.5%)", "cell_type": "PERCENTAGE", "sort_order": 74},

  {"report_id": "T-14.3.1", "execution_id": "EXEC-2026-002", "row_id": 21, "col_id": 1, "cell_value": null
    ↪ , "cell_formatted": "  Agitation", "cell_type": "TEXT", "sort_order": 75},
  {"report_id": "T-14.3.1", "execution_id": "EXEC-2026-002", "row_id": 21, "col_id": 2, "cell_value": 3, "
    ↪ cell_formatted": "3 (3.5%)", "cell_type": "PERCENTAGE", "sort_order": 76},
  {"report_id": "T-14.3.1", "execution_id": "EXEC-2026-002", "row_id": 21, "col_id": 3, "cell_value": 5, "
    ↪ cell_formatted": "5 (6.0%)", "cell_type": "PERCENTAGE", "sort_order": 77},
  {"report_id": "T-14.3.1", "execution_id": "EXEC-2026-002", "row_id": 21, "col_id": 4, "cell_value": 7, "
    ↪ cell_formatted": "7 (8.3%)", "cell_type": "PERCENTAGE", "sort_order": 78},

  {"report_id": "T-14.3.1", "execution_id": "EXEC-2026-002", "row_id": 22, "col_id": 1, "cell_value": null
    ↪ , "cell_formatted": "Source: ADAE; Population: Safety", "cell_type": "FOOTNOTE", "sort_order":
    ↪ 79},
  {"report_id": "T-14.3.1", "execution_id": "EXEC-2026-002", "row_id": 23, "col_id": 1, "cell_value": null
    ↪ , "cell_formatted": "A subject with multiple events in the same category is counted only once in
    ↪ that category. MedDRA Version 22.0.", "cell_type": "FOOTNOTE", "sort_order": 80}
]
```

# 4 Supplementary Data 4: SAP Time-to-Event Sample Text

```
Table 14.2.2: Time-to-Event Analysis of Overall Survival
A Kaplan-Meier estimate of the survival function will be computed for each treatment group. The hazard
    ↪ ratio and 95% confidence interval will be estimated using a Cox proportional hazards model with
    ↪ treatment as the only covariate. The log-rank test will be used to compare survival distributions
    ↪ between treatment groups. Censoring will be based on the CNSR variable in the ADTTE dataset. Time
    ↪ to event will be measured in days using the AVAL variable.
```

# 5 Supplementary Data 5: Legacy Library Metrics

```
# Legacy SAS Macro Library - Software Metrics Report

## Summary

| Location | File Count | Total Lines | Avg Lines/File |
|----------|-----------|-------------|----------------|
| Root (main macros) | 291 | 301,828 | 1,037 |
| adam/ (ADaM derivation) | 30 | 12,728 | 424 |
| submacros/ (helpers) | 79 | 58,142 | 736 |
| **Total** | **400** | **372,698** | **932** |

Note: The project documentation references 558 components; the count of 400 .sas files reflects that some components share files or that additional macros exist within multi-macro files (nested %macro/%mend pairs). The 558 figure counts distinct callable macros (including macros defined within other files).

## Architecture Observations

1. **Monolithic structure:** Each root-level macro typically combines data preparation, statistical computation, formatting, and rendering in a single file. Average file length exceeds 1,000 lines.

2. **High parameter count:** Representative macros have 20-50+ parameters in their %macro headers, each controlling a different aspect of behavior (input data, treatment variables, output format, titles, footnotes, display options).

3. **No separation of concerns:** Statistical logic, formatting rules, and output generation are interleaved within the same macro code blocks.

4. **Copy-paste proliferation:** Many macros are near-duplicates with minor variations (e.g., asr0ae0summary vs asr0ae0by0max0toxgr - similar structure, different grouping).

5. **No machine-readable output:** All macros produce RTF directly. There is no intermediate representation, no JSON export, and no way for external tools to programmatically inspect results.

6. **Implicit dependencies:** Macros call submacros and adam macros via implicit %include or sasautos paths. No explicit dependency declaration.

## Key Metrics for Paper

- **Total callable components:** 558 (per project documentation)
- **Total .sas files:** 400
- **Total lines of SAS code:** 372,698
- **Average lines per file:** 932
- **Largest files:** >2,000 lines (monolithic reporting macros)
- **Configuration mechanism:** Hard-coded macro parameters (no YAML/config files)
- **Output formats:** RTF only
- **AI-readable output:** None
```

# 6 Supplementary Data 6: Modernized Framework Metrics

```
# Modernized Framework ?Software Metrics Report

## Summary

| Category | File Count | Total Lines |
|----------|-----------|-------------|
| SAS macros (src/sas/) | 158 | 28,340 |
| Python modules (src/python/ + tools/) | 48 | 15,855 |
| YAML configs (config/ + registry/) | 147 | 18,825 |
| **Total** | **353** | **~63,020** |

## SAS Files by Layer

| Layer | File Count | Lines | Avg/File | Purpose |
|-------|-----------|-------|----------|---------|
| data_layer (dl_*) | 69 | 10,387 | 151 | Data preparation, subsetting, derivation |
| compute_layer (cl_*) | 43 | 11,334 | 264 | Statistical computation (21 primary + 22 specialized) |
| render_layer (re_*) | 28 | 3,139 | 112 | RTF/PDF/HTML rendering, post-processing, figures |
| adam_layer (adam_*) | 7 | 2,690 | 384 | ADaM dataset derivation and validation |
| framework (fw_*) | 9 | 790 | 88 | Logging, auditing, error handling, parameter management |
| bridge (yaml_to_sas) | 2 | -- | -- | YAML-to-SAS parameter bridging (included in framework) |
| **Total SAS** | **158** | **28,340** | **179** | |

## Python Modules

| Package | Purpose |
|---------|---------|
| orchestrator/ | Pipeline orchestration (resolver, planner, sas_generator, executor, debug_tracer) |
| ai_agent/ | SAP parser, matcher, config generator |
| infrastructure/ | SAS compute provider, storage provider |
| tools/parity_harness/ | Legacy vs native parity comparison |
| tools/migration/ | Bridge map generator, dependency analyzer, macro header parser |
| tools/esub/ | eSubmission utilities (combine TLFs, RTF-to-PDF, define.xml, etc.) |
| tools/esub_checks/ | 20-check compliance harness |
| tools/spec_to_yaml/ | ADaM/MSS spec readers |

## YAML Configuration

| Category | Count | Purpose |
|----------|-------|---------|
| Report type registry | 39 | One YAML per report type (demographics, AE summary, KM plot, etc.) |
| ADaM templates | 16 | ADaM derivation rules per dataset |
| Study configs | 25 | Per-study overrides (20 PROT008-SR1 + 5 CDISCPilot01) |
| Environment/defaults | ~10 | Runtime configuration |
| Legacy bridge map | 1 (365 entries) | 1:1 mapping legacy macro -> modernized framework |
| Schemas | 6 | JSON Schema for IR, config, registry |
| Golden RTF indices | 2 | Real-data + public parity golden references |
| **Total YAML** | **147** | |

## Key Metrics for Paper

- **Core SAS macros:** 158 files across 6 layers
- **Total SAS files:** 158
- **Total SAS lines:** 28,340
- **Average lines per SAS macro:** 179 (vs legacy avg 932 ?81% reduction)
- **Python modules:** 48 (15,855 LOC)
- **YAML configuration files:** 147 (39 report types, 18,825 LOC)
- **Bridge map:** 365 entries (1:1 legacy coverage)
- **Output formats:** RTF, PDF, HTML, JSON (vs legacy RTF-only)
- **AI-readable output:** Yes (IR -> JSON export)
- **Parity validation:** 14/14 real-data reports, 5/5 public benchmark (100% self-parity)

## Architectural Improvements

1. **Separation of concerns:** Each SAS macro has one role (data prep OR compute OR render), never
    ↪ combined.
2. **YAML-driven configuration:** Report behavior defined in YAML, not hard-coded parameters.
3. **Intermediate Representation:** Typed cell grid (ir_cells + ir_structure) decouples compute from
    ↪ render.
4. **Multi-format output:** Same IR renders to RTF, HTML, JSON, PDF.
```

```
5. **AI-cooperative:** JSON export enables LLM interaction for summarization, anomaly detection, config
    ↪ generation.
6. **Regulatory architecture:** Audit trail (fw_audit), execution manifest, electronic signatures (21 CFR
    ↪ Part 11 design).
```
